# Constitutive bone marrow adipocytes suppress local bone formation

**DOI:** 10.1172/jci.insight.160915

**Published:** 2022-11-08

**Authors:** Ziru Li, Devika P. Bagchi, Junxiong Zhu, Emily Bowers, Hui Yu, Julie Hardij, Hiroyuki Mori, Katrina Granger, Jon Skjaerlund, Gurjit Mandair, Simin Abrishami, Kanakadurga Singer, Kurt D. Hankenson, Clifford J. Rosen, Ormond A. MacDougald

**Affiliations:** 1 Department of Molecular & Integrative Physiology and; 2 Department of Orthopedic Surgery, University of Michigan Medical School, Ann Arbor, Michigan, USA.; 3 Department of Orthopedic Surgery, The Second Affiliated Hospital, Zhejiang University School of Medicine, Hangzhou, China.; 4 Department of Pediatrics, University of Michigan Medical School, Ann Arbor, Michigan, USA.; 5 Department of Biologic and Materials Sciences, School of Dentistry, University of Michigan, Ann Arbor, Michigan, USA.; 6 MaineHealth Institute for Research, Scarborough, Maine, USA.; 7 Department of Internal Medicine, University of Michigan Medical School, Ann Arbor, Michigan, USA.

**Keywords:** Bone Biology, Adipose tissue, Bone disease, Bone marrow

## Abstract

BM adipocytes (BMAd) are a unique cell population derived from BM mesenchymal progenitors and marrow adipogenic lineage precursors. Although they have long been considered to be a space filler within bone cavities, recent studies have revealed important physiological roles in hematopoiesis and bone metabolism. To date, the approaches used to study BMAd function have been confounded by contributions by nonmarrow adipocytes or by BM stromal cells. To address this gap in the field, we have developed a BMAd-specific Cre mouse model to deplete BMAds by expression of diphtheria toxin A (DTA) or by deletion of peroxisome proliferator-activated receptor gamma (*Pparg*). We found that DTA-induced loss of BMAds results in decreased hematopoietic stem and progenitor cell numbers and increased bone mass in BMAd-enriched locations, including the distal tibiae and caudal vertebrae. Elevated bone mass appears to be secondary to enhanced endosteal bone formation, suggesting a local effect caused by depletion of BMAd. Augmented bone formation with BMAd depletion protects mice from bone loss induced by caloric restriction or ovariectomy, and it facilitates the bone-healing process after fracture. Finally, ablation of *Pparg* also reduces BMAd numbers and largely recapitulates high–bone mass phenotypes observed with DTA-induced BMAd depletion.

## Graphical abstract

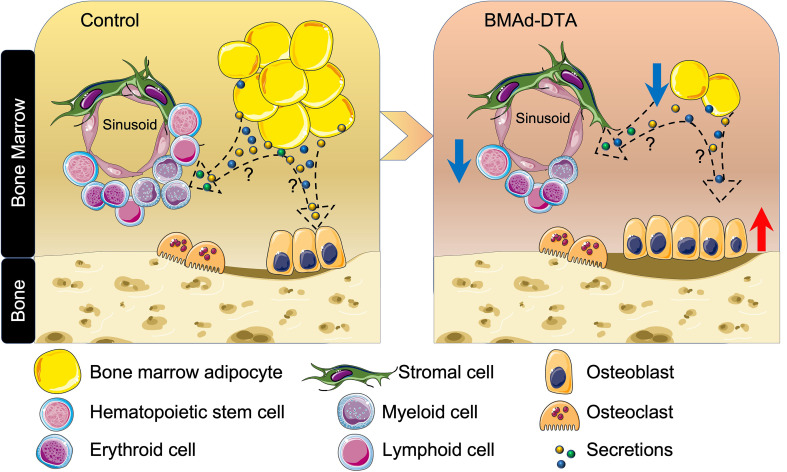

## Introduction

BM adipocytes (BMAds) are cells distinct from white and brown adipocytes that exist within BM cavities and are believed to play significant roles in hematopoiesis and bone homeostasis (1). In rodents, regulated BMAds (rBMAds) are found interspersed between hematopoietic cells in the proximal tibiae, distal femur, and lumbar vertebrae and are responsive to diverse environmental and physiological stimuli (2, 3). In contrast, constitutive BMAds (cBMAds) develop within the distal tibiae and caudal vertebrae early in postnatal life and appear relatively stable. To date, studies have shown that BM adipose tissue (BMAT) expands its population of rBMAds in response to caloric restriction (CR), ovariectomy (OVX), and irradiation, all of which are typically accompanied by bone loss and/or hematopoietic abnormalities (4, 5). Under conditions of increased energy demand or limited supply, BMAd lipolysis is required to provide energy to surrounding cells, such as osteoblasts and myeloid cells (6). However, BMAds also secrete adipokines and cytokines, which can have negative effects on bone and hematopoietic cells under both basal conditions and those that induce BMAT expansion (4, 7, 8). These seemingly conflicting results suggest that BMAds have complex functions, and further studies using highly specific models are required in order to understand the underlying mechanisms.

Other approaches used to explore the relationship between adipocytes and bone have included depletion of these cells using mice expressing diphtheria toxin A (DTA) (9) or DT receptor (DTR) (10) in all adipocytes, or adipocyte-specific deletion of genes related to adipocyte differentiation and lipid formation, such as *Pparg* (11), *Kindlin-2* (12), C*ebp,* and other bZIP family members (13), as well as *Lmna* (14). These studies suggest that loss of adipocytes, including BMAds, is associated with significant increases of bone mass; however, interpretation of these data is complicated since white, beige, and brown adipocytes, as well as BMAds, are all depleted by *Adipoq*- or *Fapb4-Cre* in these animal models. Secretion of adipokines such as adiponectin and leptin have complex effects on bone mass, with distinct and sometimes contradictory roles in osteoblast/osteoclast functions depending on whether effects are mediated peripherally or centrally, or whether the roles of adipokines on osteoblasts/osteoclasts are evaluated in vivo or in vitro (15, 16). Consistent with the notion that factors from adipose tissue play an important regulatory role in bone, Zou et al. have demonstrated that the high bone mass of lipodystrophic *Adipoq*-DTA mice is blocked by s.c. transplantation of adipocyte precursors or white or brown adipose depots, but it is only partially blocked by adipose derived from mice lacking both leptin and adiponectin (9). While important, these data do not address the local effects on bone of paracrine and metabolic factors produced by BMAds. Fan et al. revealed that local BMAT expansion promotes bone resorption through secretion of RANK ligand in a mesenchymal progenitor–specific *Pth1r*-deficient model, although interpretation of bone outcomes is complex due to mesenchymal cell fate alterations and severe growth impairment (17).

To investigate roles of BMAT in maintenance of the bone and the marrow niche, we have used a potentially novel BMAd-Cre model (6) to generate mice that specifically lack BMAT. We found that BMAd-specific DTA expression or *Pparg* deletion resulted in a significant decrease of BMAT within long bones, whereas the loss was milder in caudal vertebrae. Depletion of highly abundant constitutive BMAT (cBMAT) was accompanied by increased cortical bone in distal tibia and trabecular bone of caudal vertebrae, whereas loss of less-abundant regulated BMAT (rBMAT) resulted in mild or no effects on trabecular bone of proximal tibiae. These site-specific changes in bone mass are largely due to enhanced endosteal bone formation, which protects mice from CR- and OVX-induced cortical bone loss and facilitates high-quality bone healing after fractures. In summary, we have used BMAd-specific mouse models to establish relationships between BMAd depletion and improved bone metabolism.

## Results

### Cell-specific DTA expression depletes BMAds.

We recently created a potentially novel BMAd-specific Cre mouse model (6) that uses an intersectional strategy with dual recombinases: *Osterix-FLPo* and codon-optimized flippase recombinase (FLPo)-dependent *Adipoq-*Cre (FAC). Briefly, endogenous *Osterix*-driven FLPo recognizes Frt sites and flips the reverse orientation of the Cre cassette located in the 3′-untranslated region of endogenous *Adipoq* into the sense orientation (Supplemental Figure 1, A–C; supplemental material available online with this article; https://doi.org/10.1172/jci.insight.160915DS1). Osterix traces to osteoblasts and BMAds (18–20), and adiponectin is expressed in all mature adipocytes, including BMAds (21). Thus, the *Osterix*-FLPo–activated *Adipoq*-Cre recombinase is predominantly expressed in the overlapping BMAd population. To confirm cell specificity and efficiency of this BMAd-Cre mouse model, mT/mG reporter mice (22) were used to visualize Cre activity. EGFP^+^ cells were observed in BMAd, but not in bone cells, epididymal white adipose tissue (eWAT), or other tissues/organs evaluated (Supplemental Figure 1, D and E) (6). Of note, efficiency of BMAd-Cre increases with age, and recombination is less than 90% when mice have 2 copies of FAC and are younger than 12 weeks of age (6).

There is increasing evidence for BMAds having a specific marrow adipogenic lineage precursoe (MALP) that expresses *Adipoq, Cxcl12*, and *Lepr* (23, 24). These pericytic and stromal cells have a central cell body and have dendritic processes that extend through the marrow niche. MALPs are traced by constitutive and inducible *Adipoq*-based Cre and compose up to 0.6% of marrow cellularity (24). BMAd-Cre mice appear to target only a small subset of MALPs (Supplemental Figure 1, F–H) (6), perhaps because many are not lineage traced by *Osterix* or perhaps because expression of Cre from the endogenous *Adipoq* locus is less within this cell population relative to BMAds.

**Figure 1:**
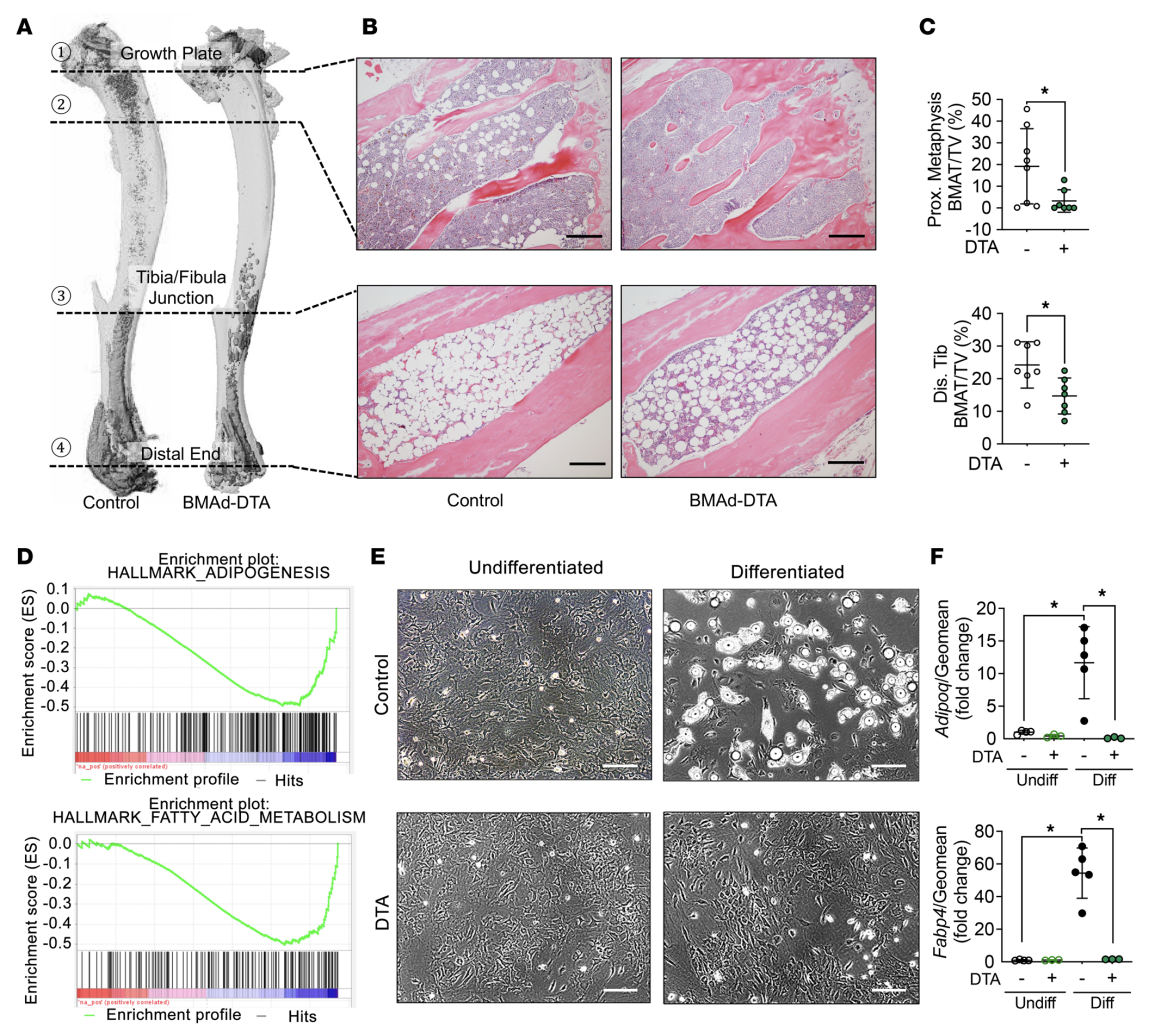
DTA expression depletes BMAds. Control (–) and BMAd-DTA (+) male mice at 20–24 weeks of age were sacrificed to validate the depletion of BMAds by DTA expression (*n* = 7 per group). Experiments were repeated more than 3 times. (**A**) Tibiae were decalcified with 14% EDTA for 3 weeks and stained with 1% osmium tetroxide for 48 hours. μCT scanning was performed, and 3D reconstituted images of tibiae are shown. (**B**) Decalcified bones were processed for paraffin embedding and sectioning. Paraffin slides were stained with H&E. Images were taken under 100× magnification. Scale bars: 200 μm. (**C**) Lipid staining within the BM was quantified by μCT scanning following osmium tetroxide staining. Proximal metaphysis is the region indicated by 1 to 2, and distal tibia is the region between 3 and 4, shown in **A**. Data are presented as mean ± SD. *P < 0.05 with a 2-tailed *t* test. (**D**) Fresh distal tibiae were collected and hammered into powder for bulk RNA purification. RNA-Seq and GSEA were performed. (**E** and **F**) BM mesenchymal stem cells were isolated from control and BMAd-DTA male mice at 16 weeks of age and were differentiated into adipocytes. Scale bar: 200 μm. After 3 weeks of differentiation, cells were collected for RNA purification, followed by qPCR. Fold changes of gene expression were normalized to the geomean of *Hprt* and *Rpl32a*. Data are presented as mean ± SD. *P < 0.05 with 2-way ANOVA with Šídák’s multiple-comparison test.

To investigate physiological functions of BMAds, we next bred BMAd-Cre mice to ROSA-DTA mice harboring a LoxP-flanked STOP cassette proximal to the DTA sequence (Supplemental Figure 1I). In this model, Cre recombinase excises the STOP cassette to allow expression of cytotoxic DTA in BMAds, thus inducing cell death. We measured BM adiposity in osmium tetroxide–stained decalcified tibiae from these mice and observed a marked reduction of rBMAT in proximal tibiae and cBMAT in distal tibiae (Figure 1A); these data were further confirmed by histological analyses of H&E-stained bone sections (Figure 1B) and quantification of osmium tetroxide by μCT (Figure 1C). Compared with control littermates, BMAd-DTA mice did not have altered total body weights, random glucose concentrations, or soft tissue weights (Supplemental Figure 2, A–C). Histological analysis of s.c. WAT (sWAT) and eWAT was comparable between BMAd-DTA and control mice (Supplemental Figure 2, D and E). Bulk RNA-Seq analysis of distal tibiae revealed that both adipogenesis and fatty acid metabolism pathways were downregulated in BMAd-DTA mice compared with controls (Figure 1D), consistent with observed reduction in BMAT content. These data were confirmed by quantitative PCR (qPCR) analysis showing that *Pparg* and *Cebpa* expression in distal tibiae are decreased in BMAd-DTA mice (Supplemental Figure 2F). We next isolated BM mesenchymal stem cells (BMSCs) and induced adipogenic differentiation. Cultures of DTA BMSCs did not have detectable lipid-containing adipocytes 21 days after induction of adipogenesis (Figure 1E). Consistent with DTA causing adipocyte cytotoxicity, adipocyte genes such as *Adipoq* and *Fabp4* were nearly undetectable in these cells (Figure 1F).

### Loss of BMAds depletes hematopoietic stem and progenitor cells (HSPCs).

 To date, studies exploring the potential role of BMAds in hematopoiesis have yielded conflicting conclusions — that BMAds impair hematopoietic regeneration or that BMAds promote hematopoiesis by secreting stem cell factors (7, 25). Our prior findings support a positive role for BMAds in hematopoieses that BMAd lipolysis is required for myelopoiesis in CR or irradiated mice (6). To determine effects of removing majority of BMAd-derived factors, we collected and analyzed whole blood from controls and BMAd-DTA mice and did not observe significant changes in circulating WBC or RBC populations. Flow cytometry analysis of hematopoietic cells isolated from long bones revealed that, although overall BM nucleated cells and T cells were decreased with loss of BMAT, most mature blood cell populations were not significantly changed (Supplemental Figure 3, A–E). In addition, HSPC populations were significantly reduced in BMAd-DTA mice, including hematopoietic stem cells (HSC), multipotent progenitors (MPP), hematopoietic progenitor cell 1 (HPC1), HPC2, premegakaryocyte/erythrocyte progenitors (PreMegE), and pre–CFU erythroid (preCFUe) precursors (Supplemental Figure 3, F–K). Furthermore, evaluation of CFU showed impaired proliferative capacity of most HSPCs isolated from tibiae of BMAd-DTA mice, including multipotential (CFU-granulocyte, erythrocyte, monocyte, megakaryocyte [GEMM]), granulocyte macrophage (CFU-GM), granulocyte (CFU-G), macrophage (CFU-M), and B lymphocyte (CFU-preB) progenitor cells, but it did not show impaired proliferative capacity of erythroid cell (CFU-E) (Supplemental Figure 3, L–Q). Interestingly, CFU-GEMM sizes were significantly smaller in BMAd-DTA mice compared with controls (Supplemental Figure 3, R and S). In support for this observation, RNA-Seq data reveal that pathways related to cell cycle, cell division, cell cycle checkpoints, DNA, and replication were extensively suppressed in BMAd-DTA mice (Supplemental Figure 3T). To investigate the mechanisms underlying alterations in these cell populations, we performed qPCR analysis on RNA isolated from sorted HSCs and MPPs and found that *c-Myc* gene expression was suppressed in MPPs derived from BMAd-DTA mice (Supplemental Figure 3, U and V). Although MYC is one of the master regulators required for hematopoietic cell growth, future studies are required to explore these mechanisms in more detail.

### BMAT depletion is accompanied by increased cortical bone formation.

**Figure 2:**
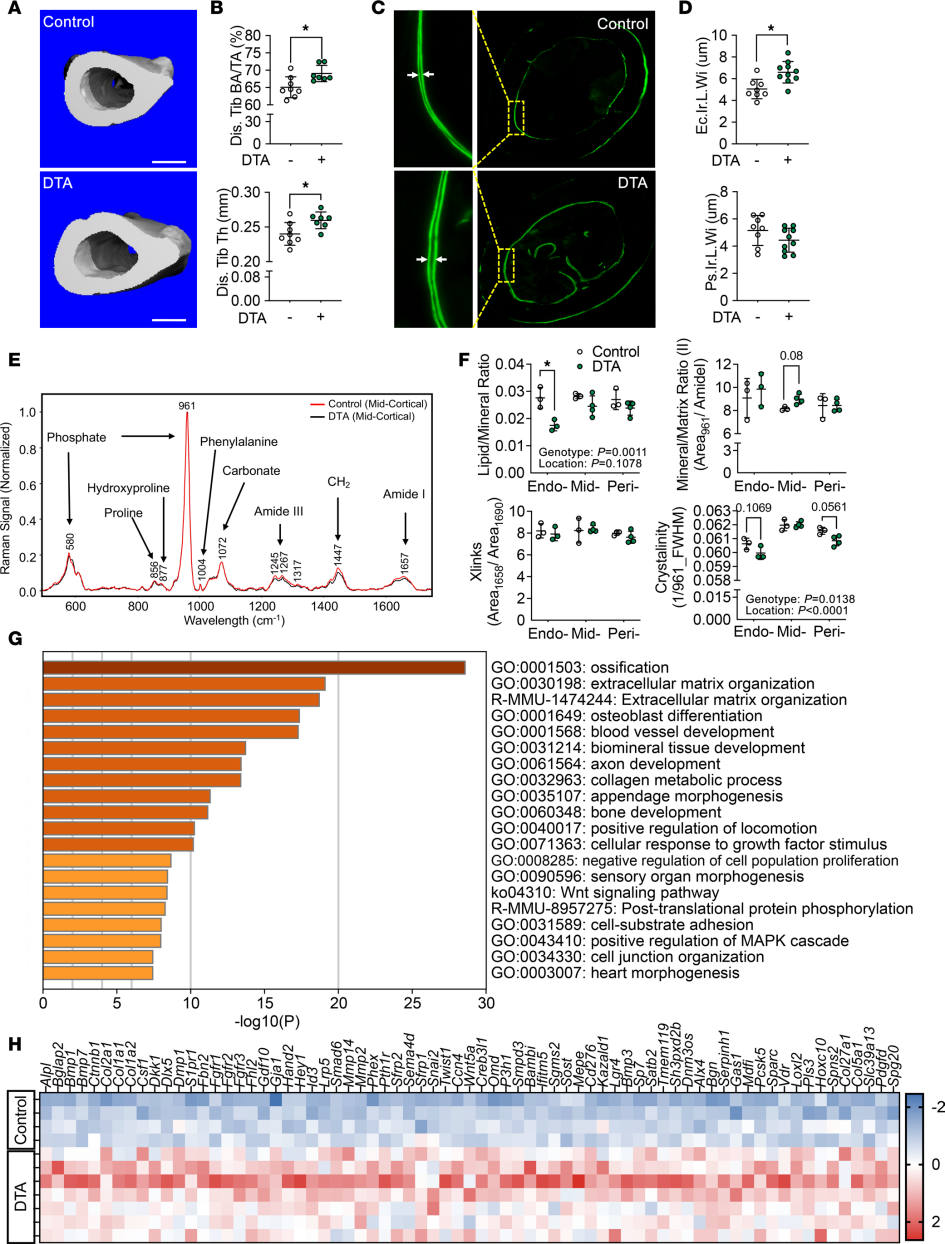
Depletion of BMAds increases cortical bone formation. Control (–) and BMAd-DTA (+) male mice at 20–24 weeks of age received calcein injections 9 and 2 days before dissection. Tibiae were collected for μCT and dynamic histomorphometry (*n* = 8–10 per group). Experiments were repeated twice. (**A**) Representative images of distal tibiae from μCT scanning are shown. Scale bar: 500 μm. (**B**) Distal tibial cortical bone area fraction (BA/TA) and thickness (Th) were quantified for control and BMAd-DTA mice. (**C** and **D**) Calcein-labeled tibiae were cross-sectioned and imaged by fluorescence microscopy. Cortical bone interlabel widths (Ir.L.Wi) in endosteum (Ec.) and periosteum (Ps.) were quantified by BioQuant software. Data are presented as mean ± SD. *P < 0.05 with a 2-tailed *t* test (**B** and **D**). (**E** and **F**) Fresh distal tibial cortical bone was cross-sectioned and analyzed with Raman microscopy (*n* = 3–4). Representative spectra from distal tibiae are shown, and major peaks are labeled (**E**). Lipid/mineral ratio, mineral/matrix ratio, collagen crosslink (Xlinks), and bone crystallinity were calculated for endosteal (Endo-), mid-cortical (Mid-), and periosteal (Peri-) regions (**F**). Data are presented as mean ± SD. *P < 0.05 with 2-way ANOVA analyses followed by Šídák’s multiple-comparison test. (**G** and **H**) Total RNA was purified from distal tibiae and used for RNA-Seq analysis (*n* = 4 for control; *n* = 7 for BMAd-DTA). The upregulated gene set was analyzed by MetaScape to identify enriched terms and pathways (**G**). *Z* scores of genes related to ossification pathway are shown as heatmap (**H**).

Given the significant changes observed within the BM niche following BMAT depletion, one might assume that development or homeostasis of bone turnover would also be affected. As mentioned above, BMAd-Cre recombinase was less efficient in BMAd-Cre mice younger than 12 weeks of age; therefore, we did not observe significant differences in length of body or long bones (Supplemental Figure 4, A–C), suggesting that bone growth remains largely unaffected; however, lumbar 5 (L5) and caudal vertebra 5 (CV5) were shorter in BMAd-DTA mice (Supplemental Figure 4D). Although loss of rBMAT in proximal tibiae was accompanied by increased trabecular bone (Tb.) volume fraction (BV/TV), mineral density (BMD), connective density (Conn. Dens), and number (N) (Supplemental Figure 4, E–G), elevated proximal tibial trabecular bone was only observed in some experimental cohorts. In contrast, higher distal tibial cortical parameters such as bone area fraction (Ct. bone area [BA]/total area [TA]) and cortical thickness (Ct. Th) were consistently found across all cohorts (Figure 2, A and B) without changes to total volume of marrow in distal tibiae (Supplemental Figure 4H). These site-specific skeletal effects may be due to the fact that there is less BMAT content in proximal tibiae than in distal tibiae. To further explore mechanisms underlying increased cortical bone mass, we performed dynamic histomorphometry analysis, which revealed significant increases in endosteal interlabel width (Ec. Ir. L. Wi) but no change in periosteum (Figure 2, C and D). Since Zou et al. previously showed that transgenic *Adipoq*-Cre-DTA mice developed osteosclerosis (9), we next used Raman microscopy to evaluate quality of endosteal, midcortical, and periosteal bone within distal tibiae (26) (Figure 2E). Consistent with loss of BMAT within the BM cavity, BMAd-DTA mice had a decreased lipid/mineral ratio in endosteal bone. Furthermore, the mineral/matrix ratio tended to be elevated in midcortical bone of BMAd-DTA mice, and crystallinity trended downward in endosteal and periosteal bone areas. Two-way ANOVA revealed a significant genotype effect, indicating that overall bone quality was improved in BMAd-DTA mice (Figure 2F). Of note, Raman collagen crosslinking (Xlinks) ratio was not different between genotypes.

To further characterize the molecular signature following BMAT depletion, we performed RNA-Seq analysis on whole distal tibiae of control and BMAd-DTA mice. We identified 841 genes that were significantly changed in BMAd-DTA mice; of these, 432 genes were increased. Pathway analysis of upregulated genes revealed that pathways related to ossification, extracellular matrix organization, and osteoblast differentiation were activated (Figure 2G). For example, further analyses of genes clustered in the ossification pathway showed that Wnt signaling–related genes (e.g., *Ctnnb1*, *Lrp4/5*, and *Wnt5a*), osteoblast markers (e.g., *Alpl*, *Bglap*, and *Bmp* genes), and osteoblast-secreted collagen genes (e.g., *Col1a1*, *Col1a2*, and *Col2a1*) were induced in BMAd-DTA mice (Figure 2H), consistent with enhancement of bone formation and mineralization (e.g., *Mepe* and *Phex*). To gain additional insights into mechanisms by which BMAd depletion enhanced bone formation, we mapped the differentially regulated genes (*P*_adj_ < 0.05) with a secretome database, MetazSecKB (27), and found that 89 genes were classified as secretory proteins. Pathway analysis of these 89 genes highlighted the induction of extracellular matrix organization and skeletal system development pathways (Supplemental Figure 4, I–K) and included factors such as *Bgn* (28), *Dmp1* (29), *Pcsk5* (30), and *Sfrp2* (31). Since bulk RNA-Seq was perform on whole distal tibiae, we were not able to distinguish specific cellular sources for the secretory factors.

### Trabecular bone formation is enhanced in caudal vertebrae of BMAd-DTA mice.

**Figure 3:**
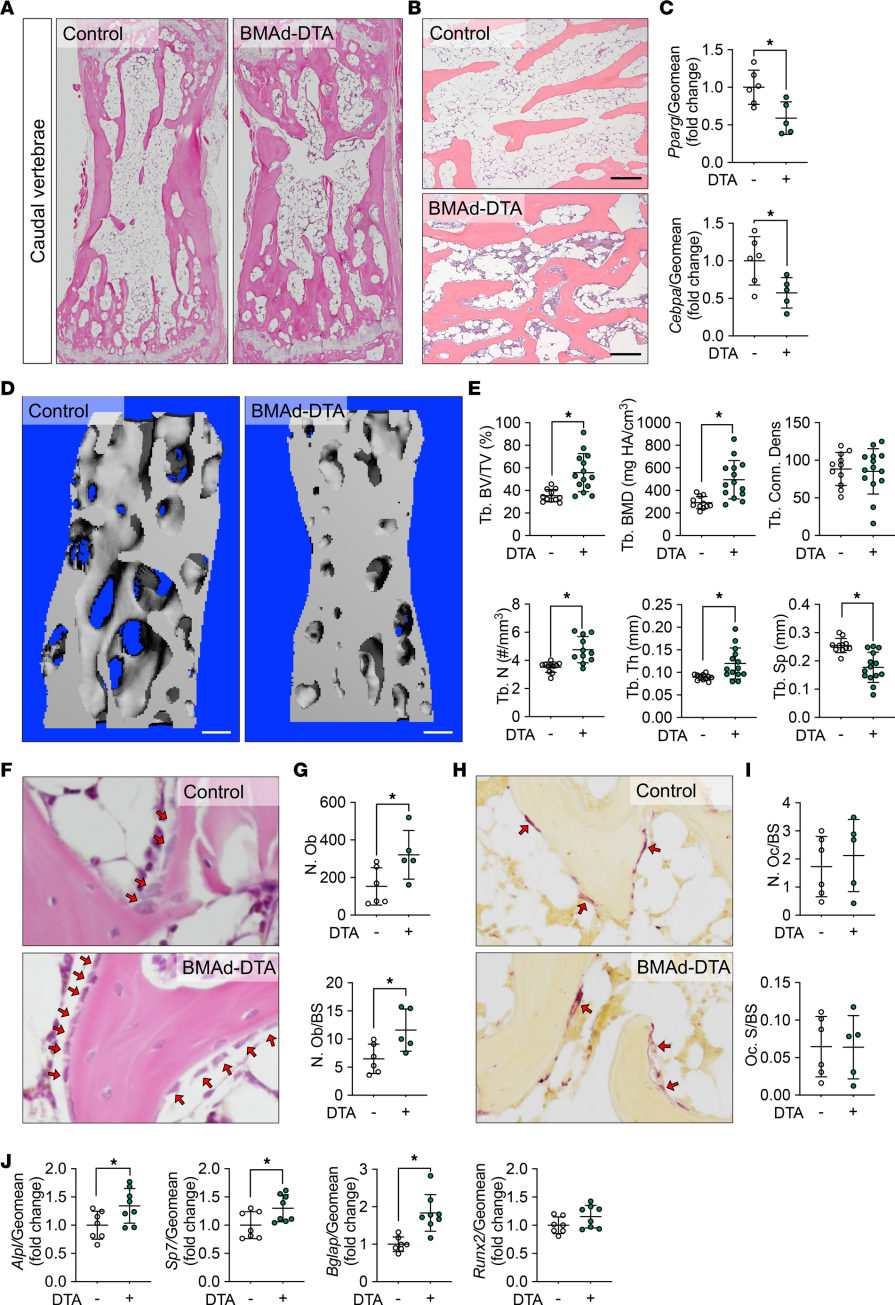
Trabecular bone formation is enhanced in caudal vertebrae of BMAd-DTA mice. Control (–) and BMAd-DTA (+) male mice at 20–24 weeks of age were sacrificed, and the fourth through sixth caudal vertebrae were collected (2 cohorts were included: one cohort with *n* = 11–14 per group was used for μCT analyses; the other cohort with *n* = 13 per group was split into histology and qPCR). (**A** and **B**) Caudal vertebrae were decalcified and used for paraffin sectioning. H&E-stained slides were scanned for an overview of the fifth caudal vertebra (**A**). Higher magnification images were taken at 100× (**B**). Scale bar: 200 μm. (**C**) RNA from the fourth through sixth caudal vertebrae was purified and used for qPCR to measure expression of adipogenic transcriptional factors. Relative gene expression is presented after normalization to the geomean of *Hprt* and *Rpl32a*. (**D** and **E**) Caudal vertebral trabecular bone parameters were assessed by CT. Scale bar: 200 μm. Tb., Trabecular bone; BV/TV, bone volume fraction; BMD, bone mineral density; Conn. Dens, connective density; N, number; Th, thickness; Sp, separation. (**F** and **G**) H&E-stained slides were used to count osteoblast number (N. Ob) and normalized to bone surface (BS). (**H** and **I**) Paraffin-sectioned slides were used for TRAP staining and osteoclast (Oc) number (N) and surface (S) quantification. (**J**) Osteogenic gene expression in caudal vertebrae were evaluated by qPCR and normalized to the geomean of *Hprt* and *Rpl32a*. Data are presented as mean ± SD. *P < 0.05 with a 2-tailed *t* test. Multiple unpaired *t* tests were performed in **E** and **J**, and *P* values were adjusted for multiple comparisons using 2-stage step-up (Benjamini, Krieger, and Yekutieli) with FDR method.

In addition to the distal tibiae, cBMAT is also enriched within caudal vertebrae (2). Interestingly, BMAd-specific DTA expression did not greatly reduce the amount of cBMAT observed within caudal vertebrae (Figure 3A). However, even with relatively minor changes in caudal cBMAT of BMAd-DTA mice, we observed increased trabecular number and larger populations of hematopoietic cells interspersed between caudal vertebral cBMAds (Figure 3B). Consistent with the notion of losing cBMAT in caudal vertebrae, expression of both *Pparg* and *Cebpa* were decreased in BMAd-DTA mice (Figure 3C), perhaps due to reduced number of cBMAd, as well as suppressed transcription factor expression per BMAd. We next performed μCT analysis, which revealed elevated Tb. BV/TV, Tb. BMD, Tb. N, and thickness (Tb. Th), and decreased trabecular separation (Tb. Sp) (Figure 3, D and E), consistent with visual observation of increased trabecular bone. Histomorphometry analysis showed an increased number of osteoblasts located on the trabecular bone surface in BMAd-DTA mice (Figure 3, F and G), whereas osteoclast number and eroded surface were not affected (Figure 3, H and I), indicating that elevated bone mass was largely due to enhanced bone formation rather than inhibited bone resorption. This conclusion is supported by expression of bone formation markers *Alpl*, *Sp7*, and *Bglap,* which were upregulated in BMAd-DTA mice compared with controls (Figure 3J).

### Loss of cBMAT protects mice from CR-induced cortical bone loss.

**Figure 4:**
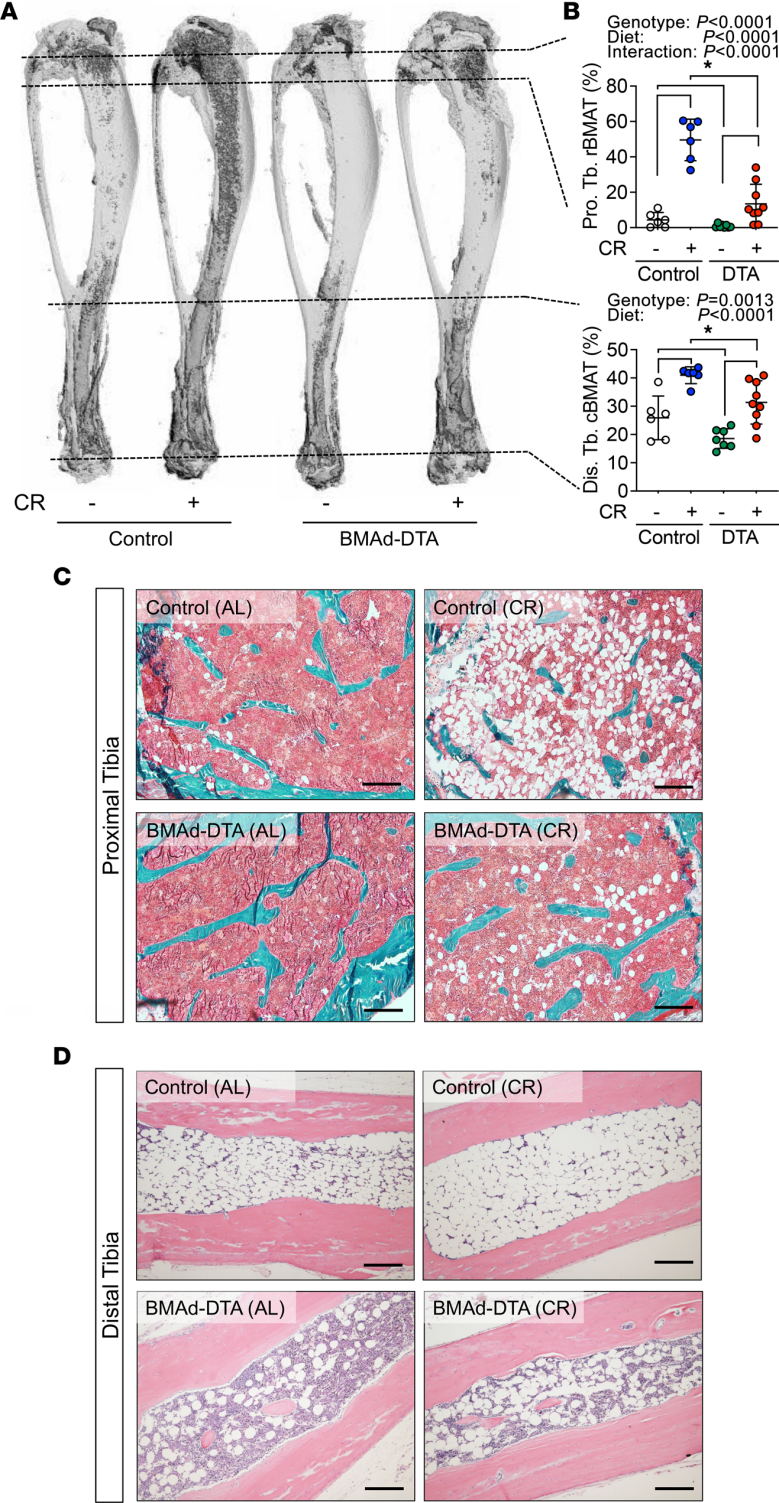
BMAd-DTA expression largely blocked caloric restriction–induced BMAT expansion. Control and BMAd-DTA male mice at 24 weeks of age underwent 30% caloric restriction (CR) for 12 weeks (*n* = 6–10 per group). Tibiae were collected for BMAT quantification. – indicates *ad libitum*, + indicates CR. (**A** and **B**) Decalcified tibiae were stained with osmium tetroxide. 3D images (**A**) and quantitative data of BMAT (**B**) were collected from μCT analyses. (**C** and **D**) Calcified (**C**) or decalcified tibiae (**D**) were plastic- or paraffin-processed and sectioned for Goldner’s trichrome (proximal tibiae; **C**) or H&E staining (distal tibiae; **D**). Images were taken under 100× magnification. Scale bar: 200 μm. Goldner’s trichrome staining, red-hematopoietic cells; green-bone, circular void-BMAds. Data are presented as mean ± SD. *P < 0.05 with 2-way ANOVA analyses followed by Šídák’s multiple-comparison test.

To determine whether enhanced bone formation observed in BMAd-DTA mice counteracts CR-induced bone loss, we challenged cohorts of mice with 30% CR for 12 weeks. As expected, following reduction of caloric supply, we observed significant reductions in body weight, glucose intolerance, and soft tissue weights, independent of genotype (Supplemental Figure 5, A–D). Additionally, s.c. and epididymal white adipocytes were much smaller in both CR-treated control and BMAd-DTA mice (Supplemental Figure 5, E and F). Consistent with previous reports (21, 32), BMAT volume was dramatically expanded in both proximal tibial rBMAT and distal tibial cBMAT of control mice following CR. Strikingly, these effects were largely blocked by DTA expression in BMAds (Figure 4, A–D).

**Figure 5:**
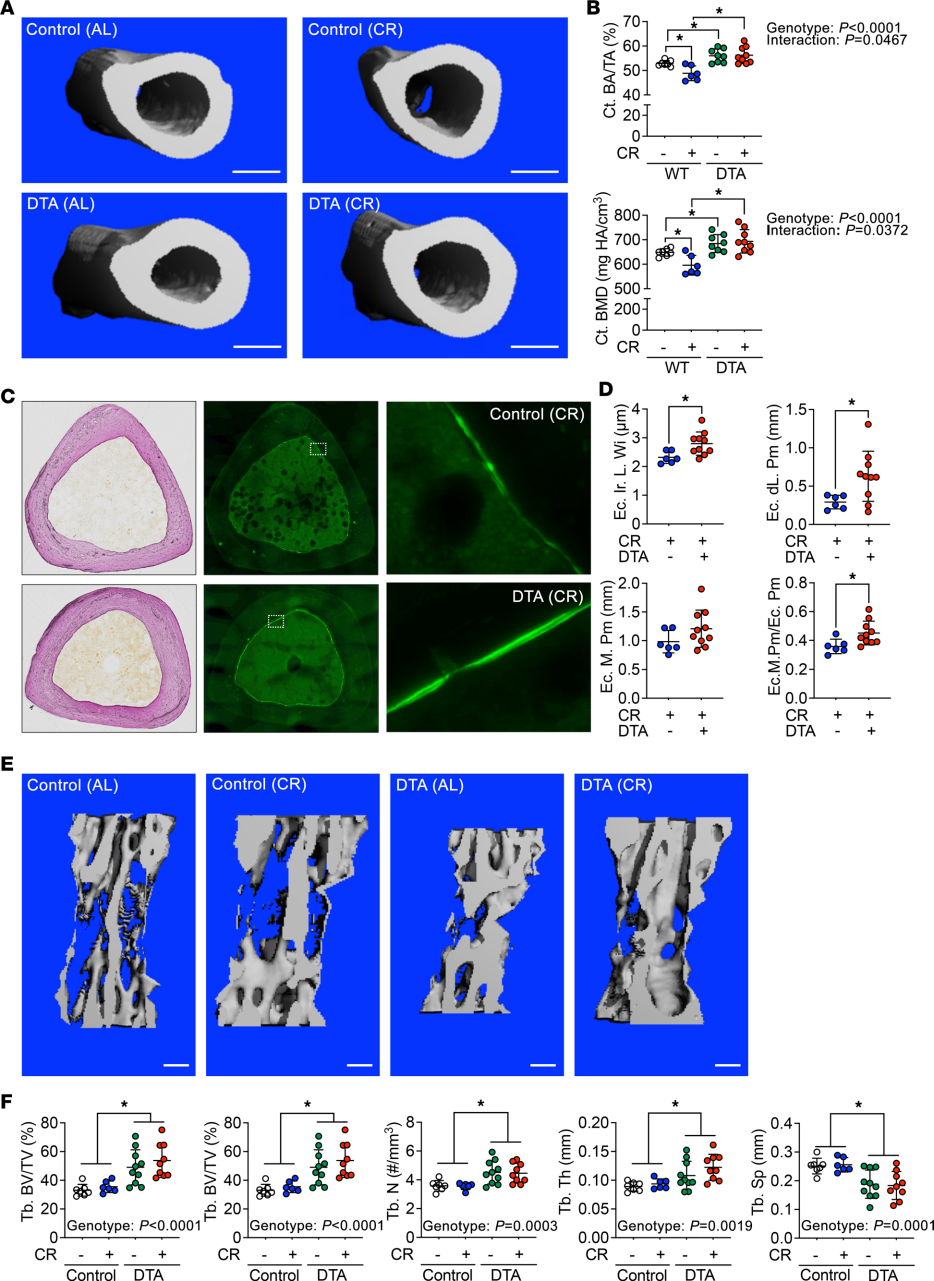
Loss of BMAds protects caloric restricted mice from loss of cortical bone. Control and BMAd-DTA male mice at 24 weeks of age underwent 30% caloric restriction (CR) for 12 weeks (*n* = 6–10 per group). Tibiae and caudal vertebrae were collected. – indicates ad libitum, + indicates CR. (**A** and **B**) Distal tibial cortical bone area fraction (Ct. BA/TA) and mineral density (Ct. BMD) were determined by μCT scanning. Scale bar: 500 μm. (**C** and **D**) Calcified distal tibiae were cross-sectioned for dynamic histomorphometry. Endosteal bone formation was measured. Ec, endocortical; Ir. L. Wi, interlabel width; dL. Pm, double-labeled perimeter; M. Pm, mineralizing perimeter. (**E** and **F**) Caudal vertebral trabecular bone variables were analyzed following μCT scanning. Scale bar: 200 μm. Tb., Trabecular bone; BV/TV, bone volume fraction; BMD, bone mineral density; Conn. Dens, connective density; N, number; Th, thickness; Sp, separation. Data are presented as mean ± SD. *P < 0.05 with 2-way ANOVA analyses followed by Šídák’s multiple-comparison test.

It is important to note that, since CR treatment was initiated in adult mice, long bone length was not affected in either genotype (Supplemental Figure 5G). Although rBMAT was dramatically enhanced by CR in control mice, CR-induced trabecular bone loss has mainly been noted at younger ages (3, 33); thus, we were not surprised to find little difference in trabecular bone variables in our cohorts (Supplemental Figure 5H). Interestingly, 12 weeks of CR in adult mice was sufficient to cause distal tibial cortical bone loss in controls, which was accompanied by a significant expansion of cBMAT toward the tibia-fibula junction. DTA expression in BMAds depleted cBMAT under standard and CR treatments and protected BMAd-DTA mice from cortical bone reduction with CR (Figure 5, A and B), without significant effects on total bone volume in distal tibiae (Supplemental Figure 5I). Dynamic histomorphometry analysis revealed that, following CR, BMAd-DTA mice had significantly increased Ir. L. Wi, double-labeled perimeter (dL. Pm), and mineralizing proportion (M. Pm/Ec. Pm), and a tending increase of mineralizing perimeter (M. Pm) at the endosteal (Ec.) bone surface (Figure 5, C and D), without affecting total endocortical perimeter (Ec. Pm) and single-labelled perimeter (sL. Pm) (Supplemental Figure 5J). In contrast, periosteal bone surfaces were minimally labeled in most CR mice, regardless of genotype (Figure 5C). In addition to enhanced endocortical bone formation, circulating bone resorption marker CTX-1 was significantly less in CR BMAd-DTA mice compared with ad libitum BMAd-DTA mice (Supplemental Figure 5K), which may partially contribute to the resistance of CR-induced cortical bone loss. CR did not affect trabecular bone parameters in caudal vertebrae from control mice; moreover, the increase of caudal vertebral bone in BMAd-DTA mice persisted, regardless of diet (Figure 5, E and F).

### cBMAT depletion protects female mice from OVX-induced cortical bone loss.

**Figure 6:**
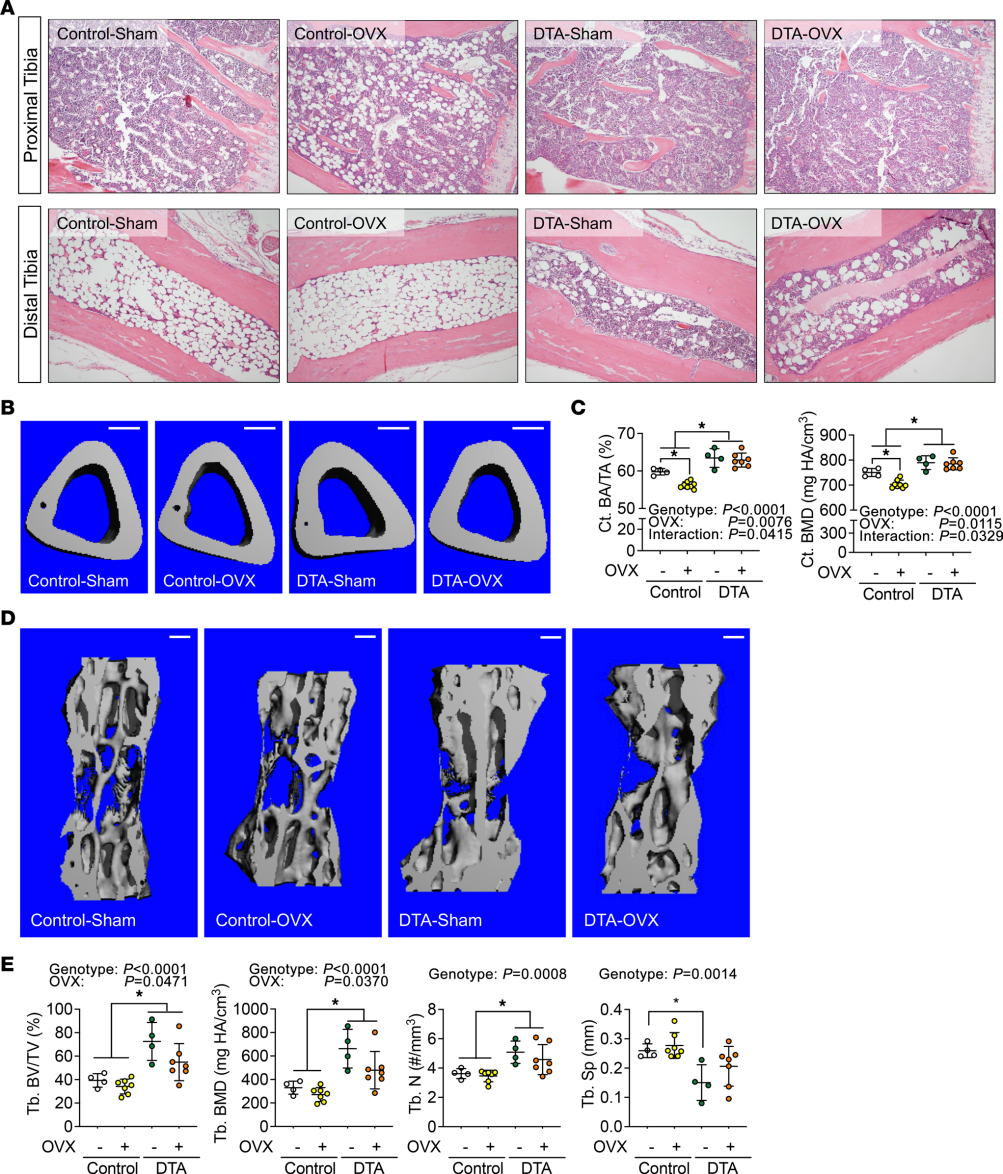
BMAd depletion protects mice from ovariectomy-induced cortical bone loss. Control and BMAd-DTA female mice at 20 weeks of age underwent ovariectomy (OVX). Mice were euthanized 6 weeks after surgery (*n* = 4–7 per group). Tibiae and caudal vertebrae were collected. – indicates sham, + indicates OVX. (**A**) Decalcified tibiae were paraffin embedded, sectioned, and H&E stained. Representative images from proximal and distal tibiae were collected under 100× magnification. Scale bar: 200 μm. (**B** and **C**) Tibiae were used for μCT scanning. Cortical bone area (Ct. BA/TA) and mineral density (Ct. BMD) at midtibia shaft were quantified. Scale bar: 200 μm. (**D** and **E**) Caudal vertebrae were scanned by CT. Trabecular bone volume (Tb. BV/TV) and mineral density (Tb. BMD), as well as microstructure parameters, were measured. Scale bar: 200 μm. Tb. N, trabecular number; Tb. Sp, trabecular separation. Data are presented as mean ± SD. *P < 0.05 with 2-way ANOVA analyses followed by Šídák’s multiple-comparison test.

To further evaluate effects of BMAT loss on bone homeostasis, female BMAd-DTA mice underwent sham or OVX surgery. Surprisingly, uterine weights were higher in BMAd-DTA mice, but OVX uniformly decreased uterine weights, as expected (Supplemental Figure 6A). We observed an effect of OVX on body weight by 3-way ANOVA, due to a small and transient increase in body weight at postoperative weeks 2 and 3 (Supplemental Figure 6B). OVX BMAd-DTA mice ultimately developed slightly impaired glucose tolerance, despite similar soft tissue weights, including sWAT, eWAT, liver, and spleen (Supplemental Figure 6, C and D). Consistent with previous studies (34, 35), OVX increased both rBMAT and cBMAT, whereas BMAd-specific DTA expression depleted BMAT at baseline and blocked OVX-induced expansion (Figure 6A). OVX stimulated a slight loss of proximal tibial trabecular bone in control and caused a significant reduction in BMAd-DTA mice (Supplemental Figure 6E). In contrast, whereas OVX induced loss of cortical bone area (Ct. BA/TA) and Ct. BMD in the middiaphysis of control mice, depletion of cBMAd increased cortical bone at baseline and protected mice from cortical bone loss with OVX (Figure 6, B and C). OVX also increased total cortical bone volume in control mice but not in BMAd-DTA mice (Supplemental Figure 6F). Although there was a trend for the circulating osteoblast marker osteocalcin to be increased by OVX and/or BMAT depletion, there were no changes in the bone resorption marker CTX-1 (Supplemental Figure 6G). Dynamic histomorphometry analyses of cortical bone revealed an increase in Ir. L. Wi in the endosteum of BMAd-DTA sham mice (Supplemental Figure 6, H and I); however, further studies will be required to explain the protective effects of BMAd-DTA mice in response to OVX. Trabecular bone of caudal vertebrae was not significantly altered by OVX in either genotype; however, BMAd-DTA mice had higher bone volume fraction (Tb. BV/TV), BMD (Tb. BMD), trabecular number (Tb. N), and tighter separation between trabeculae (Tb. Sp) when compared with controls (Figure 6, D and E). These data suggest that rBMAT and cBMAT may have opposing effects on bone homeostasis under conditions of estrogen deficiency.

### BMAT deficiency promotes bone healing.

**Figure 7:**
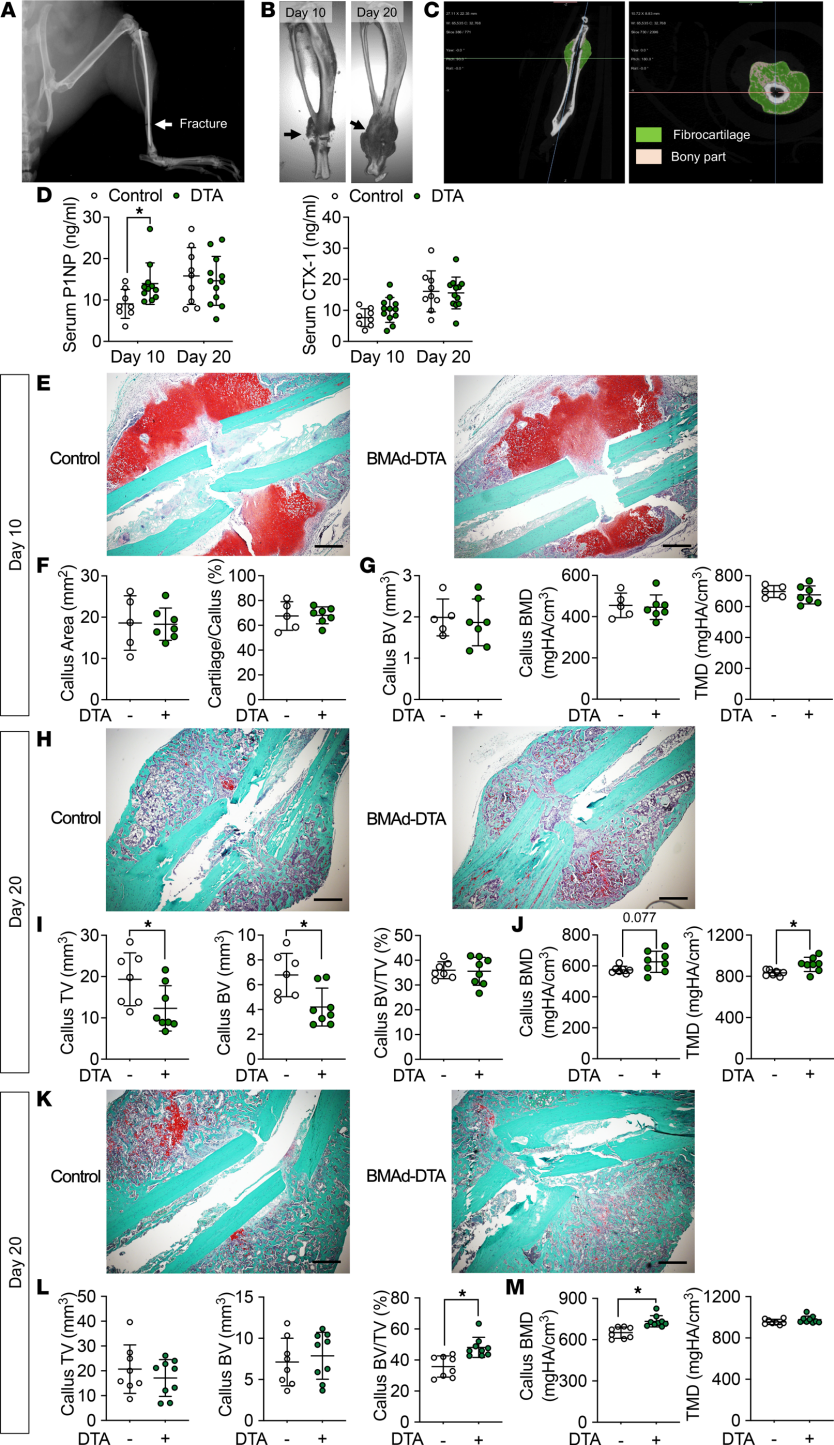
BMAd depletion promotes bone repair. (**A**–**H**) Control (–) and BMAd-DTA (+) female mice at 24 weeks of age underwent surgery to fracture distal tibiae. Tibiae and serum were collected 10 or 20 days after surgery (*n* = 12–15 per group; mice were split into day 10 or 20 euthanization after surgery). (**A**) Distal tibial fracture was visualized by x-ray scanning during surgery. (**B**) Representative 3D reconstruction images of tibial callus after 10 or 20 days of healing. (**C**) Longitudinal and cross-sectional images of callus formation from μCT analyses are shown. (**D**) Circulating bone formation marker (P1NP) and resorption marker (CTX-1) were analyzed by ELISA. Data are expressed as mean ± SD. *P < 0.05 with 2-way ANOVA analyses followed by Šídák’s multiple-comparison test. (**E**–**G**) Safranin O fast green (SOFG) staining and μCT analysis using Dragonfly software were performed to analyze callus formation in tibiae collected from day 10 of fracture healing. Green, bone tissue; orange, cartilage; dark blue, nuclei. Callus sizes and cartilage area percentage were quantified by ImageJ (NIH) (**F**). (**H**–**J**) Tibiae collected at day 20 following fracture were used for callus analysis. (**K**–**M**) Control (–) and BMAd-DTA (+) male mice at 24 weeks old underwent distal tibial fracture and were euthanized at day 20 after procedure (*n* = 8–9 per group). Callus formation was analyzed by SOFG staining and μCT scanning. TV, total volume; BV, bone volume; BV/TV, bone volume fraction; BMD, bone mineral density; TMD, tissue mineral density. Data are presented as mean ± SD. *P < 0.05 with a 2-tailed *t* test. Multiple unpaired *t* tests were performed, and *P* values were adjusted for multiple comparisons using 2-stage step-up (Benjamini, Krieger, and Yekutieli) with FDR method. Scale bar: 1 mm.

To investigate potential effects of BMAd depletion on bone repair, we induced distal tibial fractures as previously published (36) (Figure 7A). We collected tibiae at postfracture days 10 and 20 and used μCT scanning to visualize and evaluate callus formation (Figure 7, B and C). Interestingly, at day 10, the bone formation marker total procollagen type 1 N-terminal propeptide (P1NP) was increased in circulation of BMAd-DTA relative to control mice; however, P1NP concentrations were comparable between genotypes by day 20. Of note, the bone resorption marker CTX-1 remained unchanged between genotypes (Figure 7D), suggesting that BMAT depletion promotes bone formation during early stages of the fracture repair process. It was challenging to evaluate callus mineralization and bone volume fraction at postfracture day 10, given that calluses were largely composed of fibrocartilage at this time point (Figure 7, E–G). However, by postfracture day 20, fractures have mostly gone through endochondral ossification, and there is very little cartilage present. We observed a significantly smaller callus size in female BMAd-DTA mice (Figure 7, H and I). Of note, these calluses tended to have higher BMD and more dense mineralization within the bony portion (tissue mineral density [TMD]) (Figure 7J), suggesting that female BMAd-DTA mice more rapidly form higher-quality mineralized bone than control mice. In male mice, overall callus size was similar between genotypes, but BMAd-DTA mice developed higher-quality calluses with increased bone volume fraction (BV/TV) and BMD (Figure 7, K–M).

### BMAd-specific Pparg deficiency recapitulates bone phenotypes observed in BMAd-DTA mice.

**Figure 8:**
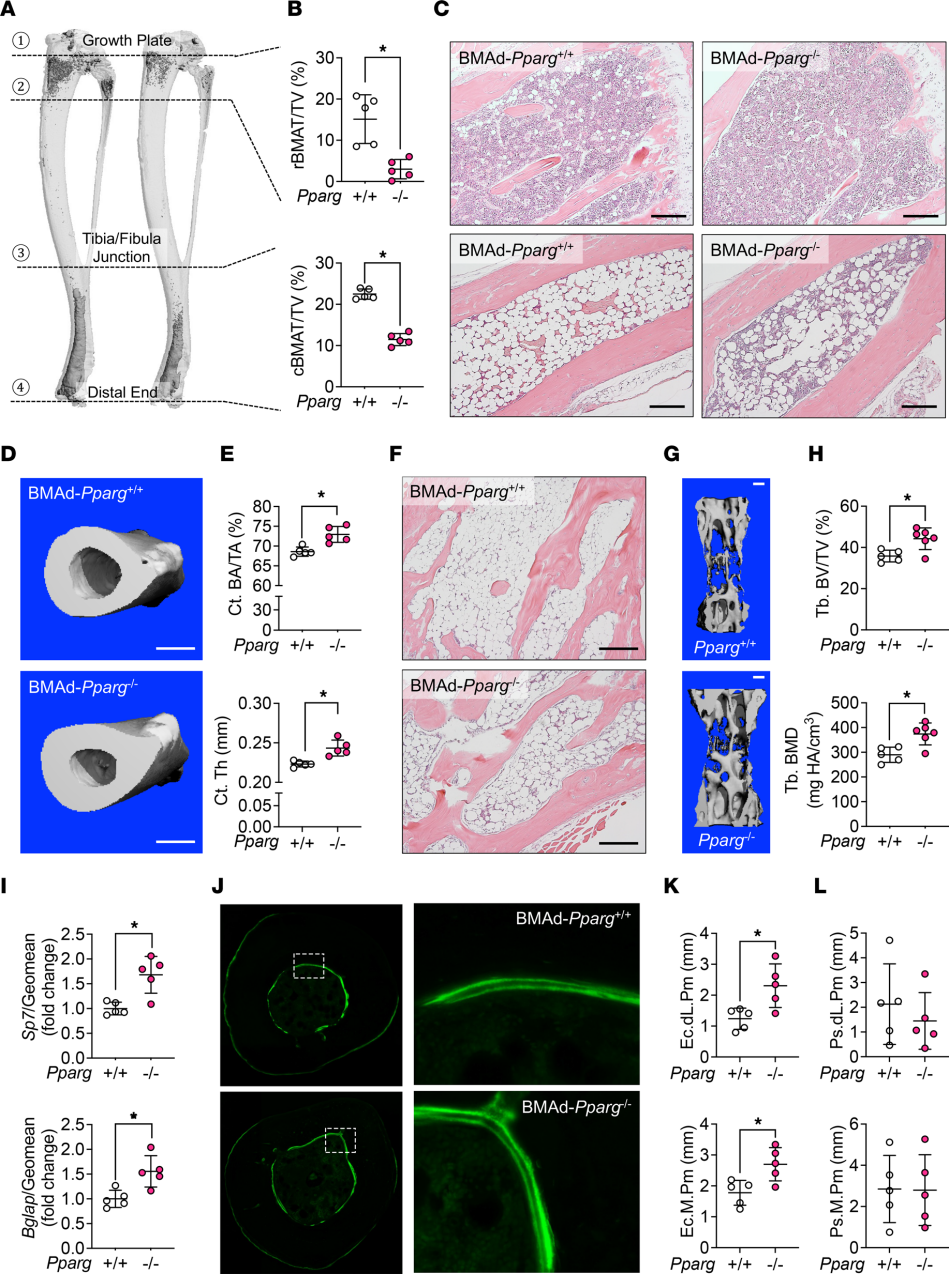
BMAd-*Pparg* deficiency recapitulates bone phenotypes observed in BMAd-DTA mice. Female mice with excision of exons 1 and 2 of *Pparg* in BMAds (BMAd-*Pparg*^–/–^) and their littermate controls (BMAd-*Pparg*^+/+^) were housed until 20 weeks of age (*n* = 5 per group). Tibiae and caudal vertebrae were collected. +/+ indicates control, –/– indicates BMAd-*Pparg* KO. (**A** and **B**) Tibiae were decalcified and stained with osmium tetroxide for 48 hours. μCT scanning was performed. Representative 3D reconstitution images of tibiae are shown (**A**). BMAT in proximal (rBMAT) and distal tibiae (cBMAT) were quantified and normalized by total volume (TV) (**B**). (**C**) Decalcified bones were processed for paraffin sectioning and H&E staining. Images were taken under 100× magnification. Scale bar: 200 μm. (**D** and **E**) Distal tibial cortical bone area (Ct. BA/TA) and thickness (Ct. Th) were quantified following μCT scanning. Scale bar: 500 μm. (**F**) Decalcified caudal vertebrae were paraffin embedded, sectioned, and H&E stained. Representative pictures were taken under 100× magnification. Scale bar: 200 μm. (**G** and **H**) Trabecular bone (Tb.) parameters in caudal vertebrae were measured by CT. Scale bar: 200 μm. BV/TV, bone volume fraction; BMD, bone mineral density. (**I**–**L**) Distal tibiae from male mice at 24 weeks of age were collected for mechanistic analyses (*n* = 5 per group). (**I**) Distal tibial total RNA was purified and used for qPCR to measure osteogenic markers. The expression of *Sp7* and *Bglap* genes were normalized to the geomean of *Hprt* and *Rpl32a*. (**J**) Distal tibiae were cross-sectioned and scanned for calcein-labeled mineralizing bone surfaces. (**K** and **L**) Dynamic histomorphometry was performed in endosteal (**K**) and periosteal (**L**) surfaces. Endocortical double-labeled perimeter (Ec. dL. Pm) and mineralizing perimeter (Ec. M. Pm) were quantified by BioQuant software. These parameters were also quantified at periosteal (Ps.) mineralizing surfaces. Data are presented as mean ± SD. *P < 0.05 with a 2-tailed *t* test.

Since DTA-induced cell death may conceivably cause tissue damage by activating inflammation or other secondary responses, we also generated a mouse model of BMAd-specific *Pparg* KO to impair adipogenesis or stimulate death of BMAd (37, 38). BMAd-*Pparg*^–/–^ female mice did not exhibit differences in body weight, random glucose concentration, or soft tissue weights (Supplemental Figure 7, A and B), whereas rBMAT and cBMAT were significantly decreased (Figure 8, A–C), confirming specificity and effectiveness of *Pparg* KO. BMAT loss did not affect bone growth, as evidenced by similar body and tibial lengths between BMAd-*Pparg*^–/–^ mice and control littermates (BMAd-*Pparg*^+/+^) (Supplemental Figure 7C). However, cBMAT-enriched distal tibiae had higher cortical bone area (BA/TA) and thickness (Th.) (Figure 8, D and E) without significant changes in total bone or marrow volume (Supplemental Figure 7D). Although the circulating bone formation marker P1NP was increased, and proximal tibial rBMAT was largely depleted, trabecular bone parameters were not influenced (Supplemental Figure 7, E and F). Similar to observations in BMAd-DTA mice (Figure 3, A and B), cBMAT was mildly decreased in caudal vertebrae of BMAd-*Pparg*^–/–^ mice (Figure 8F), and trabecular bone volume fraction (BV/TV) and BMD of caudal vertebrae were significantly higher (Figure 8, G and H). Similar results were observed in male BMAd-*Pparg*^–/–^ mice. No differences were observed for body weight, random glucose, and soft tissue weights, but an increase in trabecular bone parameters of caudal vertebrae was observed in male BMAd-*Pparg*^–/–^ mice (Supplemental Figure 8, A–F). We next used long bones of BMAd-*Pparg* male mice to further investigate mechanisms. Gene expression analysis of distal tibiae revealed upregulation of bone formation markers, such as *Sp7* and *Bglap* (Figure 8I). Dynamic histomorphometry of distal tibiae of BMAd-*Pparg*^–/–^ mice demonstrated increased double-labeled perimeter (Ec. dL. Pm), total mineral perimeter (Ec. M. Pm) and mineralizing surface per endosteal surface (Ec. M. Pm / Ec. Pm), whereas endosteal total perimeter (Ec. Pm), Ir. L. Wi, and single-labelled perimeter (Ec. sL. Pm) were not significantly changed (Figure 8, J and K, and Supplemental Figure 8G). Furthermore, these parameters were not altered on the periosteal surface (Figure 8L and Supplemental Figure 8H), providing further support for the conclusion that cBMAT depletion specifically increases endosteal bone formation.

## Discussion

In the current study, using unique mouse models, we observed functional differences in marrow adipocytes between constitutive and regulated BMAT. Previous studies have profiled the differences in size, fatty acid composition (2, 39), and lipolysis (40) between rBMAT and cBMAT; however, functional differences between these subpopulations were not well delineated. Here, we observed that rBMAT depletion in both BMAd-DTA and BMAd-*Pparg*^–/–^ mice did not consistently impact trabecular bone parameters in the proximal tibiae. On the other hand, mild and moderate reduction of cBMAT in caudal vertebrae and distal tibiae were accompanied by significant induction of trabecular and cortical bone mass, respectively. These data confirm site-specific differences in functional interactions between BMAds and osteoblasts. One possibility for this observation may be that locations enriched for cBMAT have more bone-BMAd–interacting surfaces and, thus, depletion of cBMAds has more profound effects on bone metabolism. It is also possible that cBMAds secrete distinct factors (e.g., metabolites, adipokines, extracellular vesicle cargoes) that influence local osteoblasts and bone cells through paracrine mechanisms.

One question raised in this study is why a subset of cBMAds remains within distal tibiae and caudal vertebrae in both BMAd-DTA and BMAd-*Pparg*^–/–^ models. One possibility is that the BMAd-Cre recombinase efficiency is not 100% (6). Indeed, we reported previously that BMAd-Cre efficiency was ~80% in mice at 16 weeks of age with 1 allele and increased further with age. With 2 Cre alleles, efficiency of recombination was over 90% at 12 weeks of age in mice; these observations suggest that expression of Cre might need to reach a threshold level to efficiently excise LoxP-flanked genes. Although loss of BMAds is likely to stimulate de novo adipogenesis of new adipocytes, these BMAds may not be immediately killed due to inefficiency of Cre. Another possibility is that loss of BMAds stimulates compensatory differentiation of surrounding adiponectin-negative mesenchymal stem cells into new adipocytes (41). In the *Adipoq*-driven DTA mouse model, Zhang et al. found that ectopic BMAds remained within long bones, which develop from adiponectin-negative stromal cells. Compared with adiponectin-positive BMAds, these ectopic BMAds had much lower expression of cytokines, including C-X-C motif chemokine 12 (Cxcl12), adipsin, and resistin, which may partially contribute to the impaired HSPC maintenance (42) and enhanced bone formation (43, 44) in BMAd-DTA mice.

Whereas transgenic *Adipoq*-driven expression of DTA caused severe osteosclerosis (9), we found that BMAd-specific DTA expression and *Pparg* KO had milder and healthier effects on bone formation. Indeed, Raman microscopy revealed that BMAd-DTA mice have decreased crystallinity, which is an important bone quality factor, with elevated crystallinity increasing susceptibility for osteoporotic fractures in elderly individuals (45). Moreover, increased bone mass in BMAd-DTA mice is largely due to elevated osteoblast number and enhanced bone formation. Zou et al. found that both bone formation and resorption markers were highly upregulated in *Adipoq-*DTA mice (9), indicating active bone turnover. However, since the *Adipoq*-Cre transgene targets a majority of PDGFRβ^+^/VCAM-1^+^ stromal cells, almost all Cxcl12-abundant reticular (CAR) cells (46) or MALPs (23), DTA expression driven by *Adipoq*-Cre may cause widespread damage within the BM. Interestingly, following BM injury, Cxcl12^+^ BMSCs undergo transcriptional changes that promote osteoblast-like states, associated with increased colony-forming activities and osteogenesis (47). KO with *Adipoq*-Cre of RANK ligand in MALPs and ostensibly adipocytes throughout the body including BMAds increase bone mass by suppressing turnover (48, 49). We consider an effect mediated by loss of RANK ligand and MALPs in BMAd-DTA mice unlikely, since elevated bone mass is due to increased bone formation rather than reduced bone turnover. As noted above, recombination in BMAd-specific Cre mice is restricted to BMAds and a small subset of stromal/dendritic MALPs. Our studies demonstrate that loss of BMAT is associated with increased bone formation, but the mechanisms mediating this observation remain unclear. One distinct possibility is that BMAT-derived adipokines or cytokines, such as resistin, adipsin, and IL-6, may inhibit bone formation under normal conditions. For example, global *Adipsin* KO improves bone mass following CR, thiazolidinedione treatment, and aging (43). Whole-body *IL6* deletion protects mice from trabecular bone loss and bone formation reduction caused by high-fat diet (50). In support of this notion, conditioned media from cultured adipocytes has been shown to inhibit commitment of BMSCs into the osteoblastic cell lineage in a dose-dependent manner (51). Thus, loss of BMAT and its secretome may contribute to increased osteoblast numbers and enhanced bone formation observed in BMAd-DTA and BMAd-*Pparg*^–/–^ mice.

BMAds are in close contact with hematopoietic cells (52), suggesting a significant role of these adipocytes in BM hematopoietic functions. However, BMAds have been reported to either impair (7, 8) or support (25, 53) hematopoiesis, and these contradictory outcomes may partially be due to the different mouse models that had been applied in those studies and the technical limitation of lacking cell-specific mouse model. In prior work in which BMAd lipolysis is impaired (6), BMAd-*Pnpla2*–KO mice revealed negligible hematopoietic abnormalities under basal conditions. However, with CR, proliferative capacity and postirradiation recovery of myeloid lineages was further impaired in mice lacking BMAd-*Pnpla2*. Thus, BMAds are an important energy source for myeloid lineages under these conditions. Herein, we have depleted BMAds not only as a source of energy, but also as a source of paracrine adipokines, cytokines, and other factors. We observed that BMAd depletion decreases the HSPC pool size in BM and CFU-GEMM formation and proliferation in culture. Finally, we only observed mild changes in mature hematopoietic cells in either BM or peripheral whole blood at baseline in BMAd-DTA mice; however, future studies with irradiation, aging, or transplantation may reveal additional relationships between BMAds and hematopoiesis.

In summary, we have generated 2 potentially new mouse models to investigate the functional role of BMAds within the BM niche and demonstrated that depletion of BMAT increases local bone formation and protects mice from CR- and OVX-induced bone loss. Further studies are required in order to understand the mechanisms underlying this protective role and to identify possible secretory factors that may mediate effects of BMAds on osteoblasts and other bone cells.

## Methods

### Animal

*Osterix*-FLPo, FAC, and BMAd-Cre mice were generated in a C57BL/6J and SJL mixed background by University of Michigan Transgenic Animal Model Core and MDRC Molecular Genetics Core. Mouse generation and BMAd-Cre recombinase cell specificity and efficiency were validated in our recent publication (6). mT/mG reporter (stock no. 007676), ROSA-DTA (stock no. 009669), and *Pparg^fl/fl^* mice (stock no. 004584) were purchased from The Jackson Laboratory. Since BMAd-Cre mice were shown to have hypoadiponectinemia (35), all mice used in the studies included within this manuscript were positive for both *Osterix-*FLPo and FAC. Recombination events leading to DTA expression or *Pparg* KO was determined and confirmed by genotyping across floxed alleles. Mouse genotypes were determined by PCR analysis to assess the presence or absence of floxed alleles.

#### Animal procedures.

Mice were singly housed for 2 weeks for acclimation. Mice were then maintained on a control diet (D17110202; Research Diets) for 1 week prior to beginning CR, and daily food intake was measured during this time. Mice were then fed a 30% CR diet (D19051601; Research Diets) daily at ~6:00 p.m., immediately prior to onset of the dark cycle.

Female mice at 20 weeks of age underwent OVX surgery. Mice were euthanized 6 weeks after completion of the procedure.

Distal tibial fractures were stabilized by intramedullary pins and induced by the Zondervan apparatus as previously described (36). Surgeries were performed under isoflurane anesthesia, and s.c. 0.1 mg/kg buprenorphine was given in 12-hour intervals for peri- and postoperative pain management.

### Histology and histomorphometry

Tissue histology was performed as described previously (54). Briefly, WAT depots were fixed in 10% formalin for 24 hours and subsequently embedded in paraffin for sectioning. After staining with H&E, tissues were imaged with an Olympus BX51 microscope. Tibiae were fixed in formalin for 24 hours, decalcified in EDTA for 2–3 weeks, and then fixed in 4% paraformaldehyde overnight. Bones were then embedded in paraffin and sectioned. Bones were stained with H&E or tartrate-resistant acid phosphatase (TRAP) as indicated, and slides were scanned at 200× magnification. Static measurements, include trabecular bone surface (BS), osteoblast number (N. Ob), and osteoclast number (N. Oc) and eroded surface (Oc. S), were evaluated on H&E or TRAP staining images. Undecalcified tibiae and femurs were used for plastic embedding and cross-sectioning to visualize endosteal and periosteal regions of cortical bones. Cortical bone area and endosteal and periosteal perimeters were evaluated with Goldner’s Trichrome Staining. For dynamic studies, calcein (C0857; Sigma-Aldrich) dissolved in 0.02 g/mL sodium bicarbonate with 0.9% saline at 20 mg/kg was injected i.p. at 9 and 2 days prior to sacrifice for quantification of mineralizing surface and Ir. L. Wi of cortical bone. Calculations were performed using Bioquant Osteo 2014 software in a blinded manner (55, 56).

### μCT analysis

Tibiae were placed in a 19 mm diameter specimen holder and the entire bone length was scanned using a μCT system (CT100 Scanco Medical). Scan settings were as follows: voxel size 12 μm, 70 kVp, 114 μA, 0.5 mm AL filter, and integration time 500 ms. Density measurements were calibrated to the manufacturer’s hydroxyapatite phantom. Analysis was performed using the manufacturer’s evaluation software with a threshold of 180 for trabecular bone and 280 for cortical bone. Trabecular bone analysis was performed on 0.6 mm of tibia and initiated 60 μm distal from growth plate. Distal tibial cortical bone analysis was performed between tibia/fibula junction and distal end.

### Marrow adiposity quantification by osmium tetroxide staining and μCT

Mouse tibiae were decalcified for 2–3 weeks before osmium tetroxide staining, as previously described (2, 57). A threshold of 300 greyscale units was used for proximal tibial rBMAT quantification, given the smaller adipocyte size and, thus, lower density of osmium staining. A threshold of 400 greyscale units was used for distal tibial cBMAT. Representative 3D images were exported from the CT100 Scanco Medical platform.

### Raman microscopy and data analysis

Bone composition analyses of fresh distal tibial specimens were performed using a Raman microscope equipped with a near-infrared laser 785 nm diode laser (Innovative Photonics Solutions) and a 25 μm slit to give a spectral resolution of ~4 cm^–1^, as previously described (58). The excitation laser was spot-focused through a 10×/0.50 NA objective (S Fluor; Nikon Instruments Inc.) to give ~40 mW of laser power at the specimen surface. The spatial resolution of the Raman microscope was determined to be ~2.8 μm as defined by the length of 2 pixels of Raman images obtained from a negative USAF 1951 test target (Edmond Scientific, Group-7; Element-6) (59, 60). Distal ends of tibial specimens were embedded upright in polymethylmethacrylate (Koldmount Cold Mounting Kit, Mager Scientific) (61), followed by light polishing of the proximal ends on silicon carbide paper (Grit 4000, Buehler) and rinsing with deionized water. Cortical surface of proximal ends were brought into focus under the Raman microscope stage, and a series of spectra were acquired from endosteal, midcortical, and periosteal surfaces at the lateral, medial, anterior, and posterior quadrants (58). Periosteal and endosteal measurement sites were defined as ~5 μm from inner and outer perimeters of cortical sections. This approach ensured that spectra were acquired with minimal interference from residual nonosseous tissue at the periosteum, blood, and lipid residues at the endosteal bone/medullary interface (58). A total of 12 cortical spectra were acquired from each specimen using a spectral accumulation time of 6 minutes.

Spectra were processed in MATLAB (The MathWorks Inc.) and imported into GRAMS/AITM Software (Thermo Fisher Scientific) for baseline correction, normalization, and curve-fit analysis (58). Select band area ratios were calculated for the following compositional traits: mineral/matrix ratio (~961/1,659 cm^–1^); XLinks ratio (~1,659/1690 cm^–1^), and lipid/mineral ratio (~1,299/961 cm^–1^) (58, 62). Mineral/matrix ratio is related to the amount of mineral within a given volume of bone matrix analyzed and is positively correlated with TMD (61). The XLinks ratio reflects the relative ratio of the mature trivalent (pyridinoline) crosslink to the immature divalent (dehydrodihydroxylysinonorleucine) crosslink. The lipid/mineral ratio is a variant of the lipid/matrix ratio, which has been shown to increase positively with age in human male radii (62). Mineral crystallinity is the inverse of the full-width at half maximum (1/FWHM) of the Gaussian-fitted _v1_PO4 band at ~961 cm^–1^ and is a measure of bone mineral crystallite size and/or lattice perfection (63, 64). Results were averaged for endosteal, midcortical, and periosteal bone surfaces.

### RNA extraction and qPCR

Distal tibiae or caudal vertebrae were powdered in liquid nitrogen and lysed in RNA Stat-60 reagent in a precooled dounce homogenizer to extract total RNA. qPCR was performed using an Applied Biosystems QuantStudio 3 qPCR machine. Gene expression was calculated based on a cDNA standard curve within each plate and normalized to expression of the geometric mean of housekeeping genes *Hprt* and *Rpl32a*.

### Bulk RNA-Seq

Total RNA was isolated from distal tibiae for strand-specific mRNA sequencing (Beijing Genomics Institute). Over 20 million reads were obtained using a paired-end 100 bp module on DNBSEQ platform. Quality of raw reads were checked using FastQC (v.0.11.9), and filtered reads were aligned to a reference genome (UCSC mm10) using STAR with default parameters. All samples passed the postalignment quality check (QualiMap, v.2.2.1). The DEseq2 method was used for differential gene expression analysis with genotype (DTA control versus) as the main effect. Pathway analysis was conducted on ranked lists by the metric −log_10_ FDR × log_2_ fold change. The resulting list was run through preranked gene set enrichment analysis (GSEA) using the Molecular Signatures Database v7.1 (H, hallmark gene sets), as previously described (65). Gene ontology analysis was performed using MetaScape. Heatmaps were generated using the R pheatmap package, and the complete-linkage clustering method was used to hierarchically cluster genes. These data are available through NCBI GEO with accession no. GSE199973.

### Flow cytometry

Tibiae were isolated from mice, and BM was harvested by flushing with 1 mL ice-cold PEB (1× PBS with 2 mM EDTA and 0.5% BSA). RBCs were lysed once using 1 mL RBC Lysis Buffer (155 mM NH_4_Cl, 10 mM KHCO_3_, 0.1 mM EDTA). Cells were immediately pelleted by centrifugation at 300 x g for 5 minutes at room temperature and resuspended in 1 mL ice-cold PEB. Staining and flow cytometry gating strategies were performed as previously described (6).

### CFU assay

BM cells from control and BMAd-DTA mice were isolated from tibia. Bones were flushed with IMDM (Thermo Fisher Scientific, 12440) containing penicillin-streptomycin antibiotic (Thermo Fisher Scientific, 15270-063), and BM cells dispersed using trituration. Cells were pelleted and counted with a hemocytometer. After 1 × 10^4^ cells were plated in MethoCult medium (Stemcell Technologies, M3434) in 35 mm culture dishes, cells were incubated at 37°C in 5% CO_2_ with ≥ 95% humidity for 7 days. CFU-GEMM, CFU-GM, CFU-G, CFU-M, CFU-E, CFU-preB, and colonies on each plate were counted using a microscope with a 4× objective lens.

### Bone callus analysis

Whole tibiae were collected for μCT scanning 10 or 20 days after distal tibial fracture surgery. After 3D reconstruction, newly formed calluses were quantified using Dragonfly software, as described (66). Briefly, using 3D and 2D images, callus and cortical bone sections were identified manually, and then spline interpolation was performed between slices no more than 10 slices apart (0.105 mm). Points were reviewed and reinterpolation was performed as necessary. The Otsu method (https://ieeexplore.ieee.org/document/4310076; doi:10.1109/TSMC.1979.4310076) as implemented in Dragonfly (version 2021.1) was used to threshold the image into bone and background. Cortical bone sections were removed, and an adjusted Otsu threshold was applied to calculate bone volume, TMD, callus volume, and BMD throughout the whole cohort.

### BMSC isolation and differentiation

Femurs and tibiae were collected from BMAd-DTA mice and control littermates, and BMSCs were isolated as previously described (67). After 48 hours, adherent BMSCs were further proliferated until 2 days after confluence. Cells then underwent induction of adipogenesis as previously published (65).

### Statistics

Significant differences between 2 groups were assessed using a 2-tailed *t* test, and *P* values were adjusted for multiple comparisons using 2-stage step-up (Benjamini, Krieger, and Yekutieli) with FDR method. Two-way ANOVA with Šídák’s multiple-comparison test was performed for data presented in Figure 4, Figure 5, and Figure 6, as well as in related Supplemental Figures 5 and 6. Three-way ANOVA with Šídák’s multiple-comparison test was performed for data presented in Supplemental Figure 6, B and C. Image J software (NIH) was used to analyze the callus sizes and cartilage area percentage in Figure 7. All analyses were conducted using GraphPad Prism version 9. All data are presented as mean ± SD. For statistical comparisons, *P* < 0.05 was considered to be significant.

### Study approval

Mice were housed in a 12-hour light/dark cycle in the Unit for Laboratory Animal Medicine at the University of Michigan, with free access to water. Mice were fed ad libitum or underwent CR, as indicated. All procedures were approved by the University of Michigan Committee on the Use and Care of Animals.

## Author contributions

ZL, KS, KDH, CJR, and OAM conceived the studies and planned the experimental design. ZL, DPB, JZ, EB, JH, HM, KG, JS, GM, and SA performed the experiments. ZL, HY, and OAM analyzed the data. ZL, DPB, and OAM wrote the manuscript, while all other authors edited and approved the final manuscript.

## Supplementary Material

Supplemental data

## Version 1 09/01/2022

In-Press Preview

## Version 2 11/08/2022

Electronic publication
