# Supplementary material for: Constitutive bone marrow adipocytes suppress local bone formation
Source: JCI Insight. 2022 Nov 8;7(21):e160915. doi: 10.1172/jci.insight.160915 (PMC9675472; doi:10.1172/jci.insight.160915)
Supplement: Supplemental data [file jciinsight-7-160915-s162.pdf]

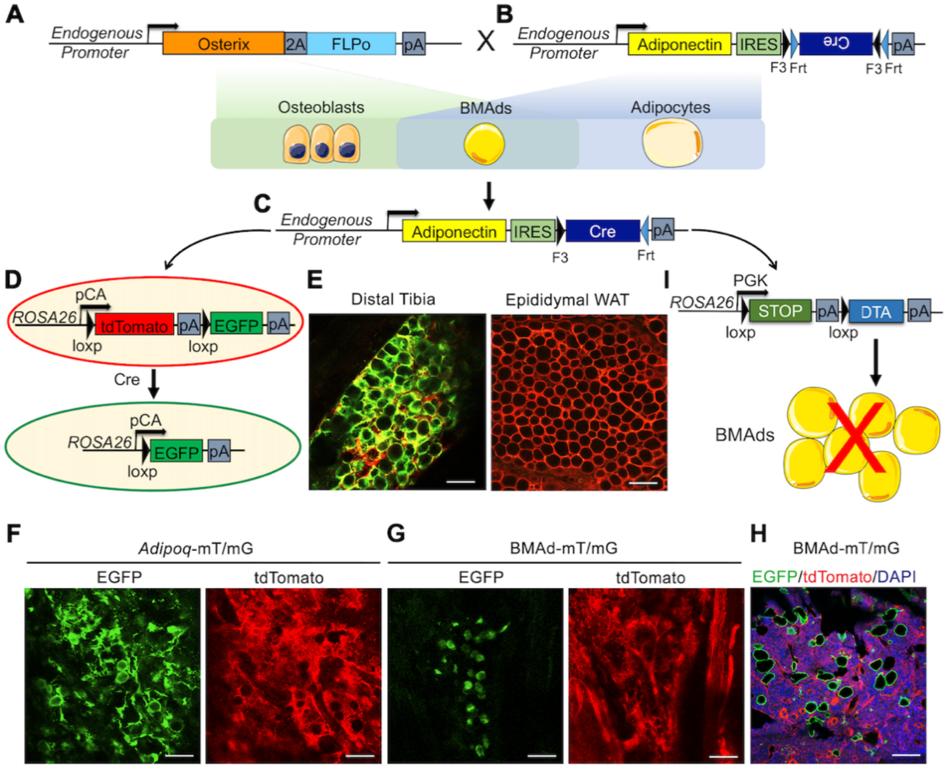

Supplemental Figure 1. Schematic of bone marrow adipocyte (BMAd)-specific mouse model.
A. Gene structure of *Osterix*-FLPo. A FLPo-polyA cassette was inserted in the 3'-UTR of endogenous *Osterix* gene. The resulting Osterix-FLPo fusion protein is divided by a 2A self-cleavage peptide.
B. Schematic of FLPo-activated *Adiponectin* Cre (FAC). IRES and F3/Frt-flanked Cre cassette in the reverse direction were inserted in the 3'-UTR of endogenous *Adipoq*.
C. Schematic model of BMAd-specific mice. When *Osterix*-FLPo mice are bred with FAC mice, the Cre cassette in the 3'-UTR of endogenous *Adipoq* is recombined by FLPo to the correct direction, and is thus predominantly expressed in the overlapping cell population, BMAd.
D-E. Validation of specificity and efficiency of BMAd-Cre recombinase.
D. Progeny of BMAd-Cre and mT/mG reporter mice express membrane EGFP only in cells that have expressed Cre.
E. Male mice at 20 weeks of age were sacrificed and fresh tissues were analyzed. Tibiae were bisected longitudinally by razor blade to expose BMAT. BMAT of distal tibiae and a thin piece of epididymal WAT were directly evaluated by confocal microscopy, and representative images are presented.
F. To visualize *Adipoq* traced bone marrow adipocytes and stromal cells, *Adipoq*-Cre mice were bred with mT/mG reporter. Proximal tibial fresh confocal was performed when mice were 16 weeks old.
G-H. BMAd-Cre mice were bred with mT/mG reporter mice resulting in BMAd-mT/mG mice, which were euthanized at 20 weeks old. Fresh tissue confocal (G) and frozen sections (H) were used to determine the cell-specificity of BMAd-Cre expression.
I. Model of BMAd-DTA mice. BMAd-Cre mice were bred with ROSA-DTA mice, which harbor a LoxP-flanked STOP cassette proximal to a DTA cassette. Excision of the STOP cassette by Cre is necessary for DTA expression in BMAds to induce cell death.

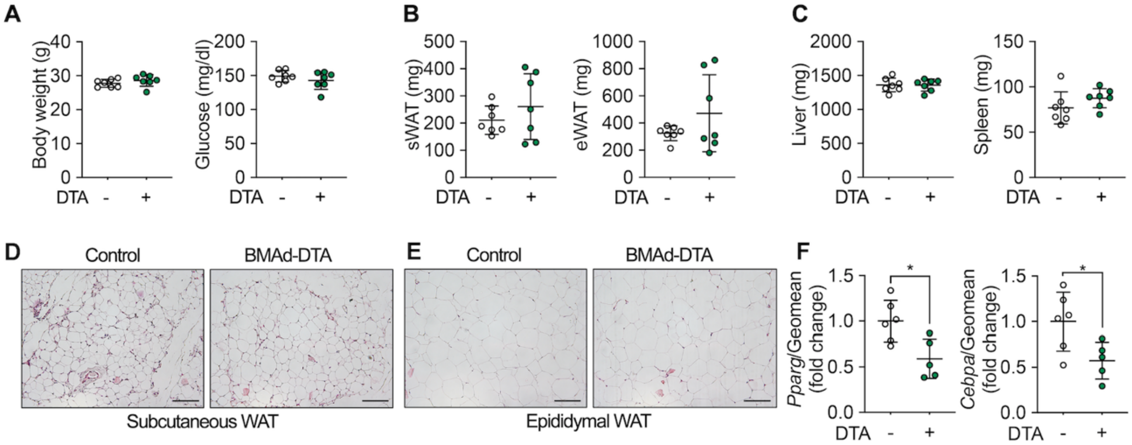

Supplemental Figure 2. BMAd-specific DTA expression does not cause a detectable phenotype beyond bone and the marrow niche.
Control (-) and BMAd-DTA (+) male mice were sacrificed at 20-24 weeks of age. Experiments were repeated more than three times.
A-C. Body weights, random glucose concentrations and soft tissue weights were measured at the end of the experiment.
D-E. Images of subcutaneous and epididymal WAT depots were taken under 200X magnification following paraffin sectioning and H&E staining. Scale bar indicates 100 μm.
F. RNA purified from distal tibiae was used for qPCR to measure lipogenic gene expression, which was normalized to the geomean of *Hprt* and *Rpl32a*.
Data are presented as mean ± SD. * indicates $P < 0.05$ with a two-sample *t*-test.

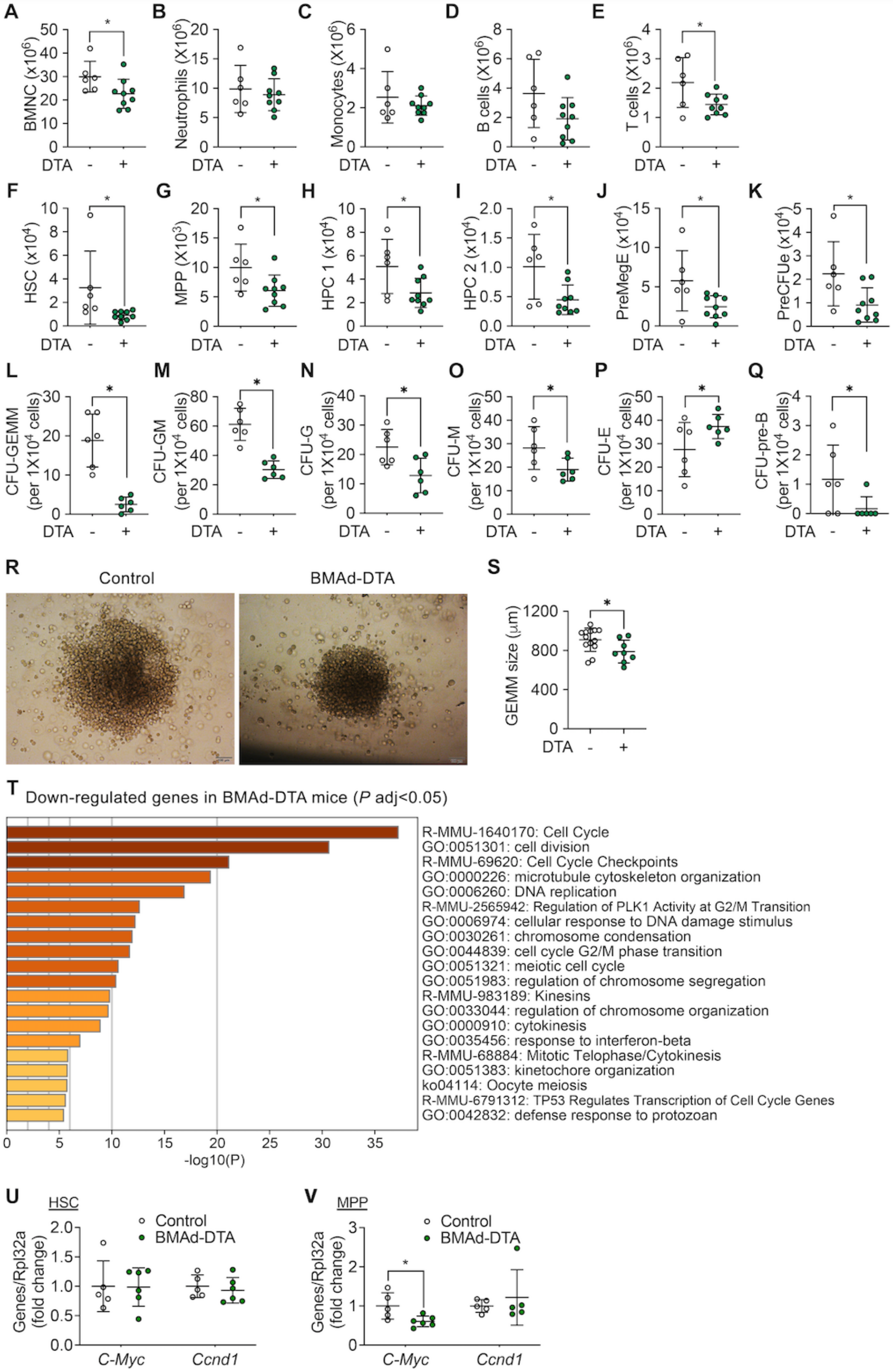

A
B
C
D
E
BMNC (x10^6)
Neutrophils (X10^6)
Monocytes (X10^6)
B cells (X10^6)
T cells (X10^6)
DTA - +
F
G
H
I
J
K
HSC (x10^4)
MPP (X10^3)
HPC 1 (x10^4)
HPC 2 (x10^4)
PreMegE (x10^4)
PreCFUe (x10^4)
L
M
N
O
P
Q
CFU-GEMM (per 1X10^4 cells)
CFU-GM (per 1X10^4 cells)
CFU-G (per 1X10^4 cells)
CFU-M (per 1X10^4 cells)
CFU-E (per 1X10^4 cells)
CFU-pre-B (per 1X10^4 cells)
R
Control
BMAd-DTA
S
GEMM size (μm)
T
Down-regulated genes in BMAd-DTA mice (P adj<0.05)
R-MMU-1640170: Cell Cycle
GO:0051301: cell division
R-MMU-69620: Cell Cycle Checkpoints
GO:0000226: microtubule cytoskeleton organization
GO:0006260: DNA replication
R-MMU-2565942: Regulation of PLK1 Activity at G2/M Transition
GO:0006974: cellular response to DNA damage stimulus
GO:0030261: chromosome condensation
GO:0044839: cell cycle G2/M phase transition
GO:0051321: meiotic cell cycle
GO:0051983: regulation of chromosome segregation
R-MMU-983189: Kinesins
GO:0033044: regulation of chromosome organization
GO:0000910: cytokinesis
GO:0035456: response to interferon-beta
R-MMU-68884: Mitotic Telophase/Cytokinesis
GO:0051383: kinetochore organization
ko04114: Oocyte meiosis
R-MMU-6791312: TP53 Regulates Transcription of Cell Cycle Genes
GO:0042832: defense response to protozoan
-log10(P)
U
HSC
V
MPP
Control
BMAd-DTA
Genes/Rpl32a (fold change)
C-Myc
Ccnd1

Supplemental Figure 3. BMAT depletion results in loss of hematopoietic stem and progenitor cells (HSPCs).

Control (-) and BMAd-DTA (+) male mice were sacrificed at 20-24 weeks of age. Experiments were repeated twice.

A-E. Mature hematopoietic cell counts from flow cytometry are shown (n=6-9 per group). BMNC: bone marrow mononuclear cell.

F-K. Bone marrow cells were isolated and stained for flow cytometry to analyze the HSPCs. HSC: hematopoietic stem cell; MPP: multi-potent progenitor; HPC: hematopoietic progenitor cell; PreMegE: pre-megakaryocyte-erythrocyte progenitor; PreCFUe: erythrocyte progenitor (pre-colony forming unit-erythrocyte).

L-S. CFU assays. Tibial bone marrow cells were isolated from BMAd-DTA mice and littermate controls. After counting, $1x10^4$ cells were plated for CFU assays. Colonies were counted by an independent expert in a blinded manner at 10 days after plating (L-Q). GEMM: granulocyte, erythroid, macrophage, megakaryocyte; GM: granulocyte, macrophage; G: granulocyte; M: macrophage; E: erythroid; pre-B: B-lymphoid progenitors. Representative images of CFU-GEMM; scale bar indicates 100 μm (R). GEMM colony sizes were quantified by Image J (S).

T. Fresh distal tibiae were collected and hammered into powder for bulk RNA purification. RNA-seq and pathway analysis were performed.

U-V. Sorted HSCs and MPPs were processed for RNA isolation. qPCRs were performed to measure expression of proliferation-related genes, *C-Myc* and *Ccnd1*. Relative gene expression is shown after normalization to *Rpl32a*.

Data are presented as mean ± SD. * indicates $P < 0.05$ with a two-sample *t*-test. Multiple unpaired *t*-tests were performed, and *P* values were adjusted for multiple comparisons using two-stage step-up (Benjamini, Krieger, and Yekutieli) with FDR method.

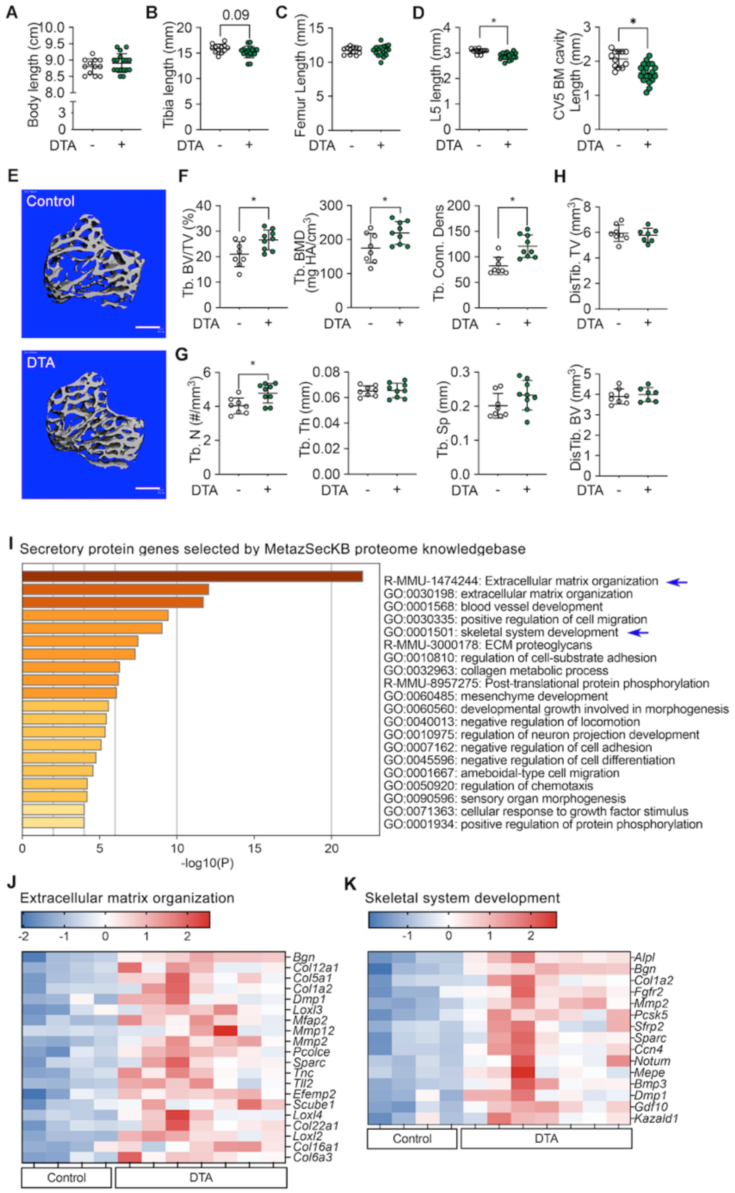

Supplemental Figure 4. BMAd-depletion does not affect long bone growth, but shortens lengths of vertebrae.

Control (-) and BMAd-DTA (+) male mice at 20-24 weeks of age were sacrificed, and tibiae, femurs and caudal vertebrae collected. Experiments were repeated twice.

A-D. Length of body from nose to base of tail, tibia, femur, lumbar vertebra 5 (L5) and caudal vertebra 5 (CV5).

E-G. Proximal tibial trabecular bone parameters were determined by μCT scanning. Scale bar indicates 500 μm. Tb. Trabecular bone; BV/TV: bone volume fraction; BMD: bone mineral density; Conn. Dens: connective density; N: number; Th: thickness; Sp: separation.

H. Distal tibial total volume (TV) and bone volume (BV) were measured by μCT.

Data are presented as mean ± SD. * indicates $P < 0.05$ with a two-sample *t*-test. Multiple unpaired *t*-tests were performed, and *P* values were adjusted for multiple comparisons using two-stage step-up (Benjamini, Krieger, and Yekutieli) with FDR method.

I. Differential genes regulated by BMAd-DTA in distal tibiae were mapped with MetaZSecKB secretome datasets. Secretory protein genes were used for pathway analyses.

J-K. Z-Scores of secretory protein genes enriched in extracellular matrix organization (J) and skeletal system development (K) pathways.

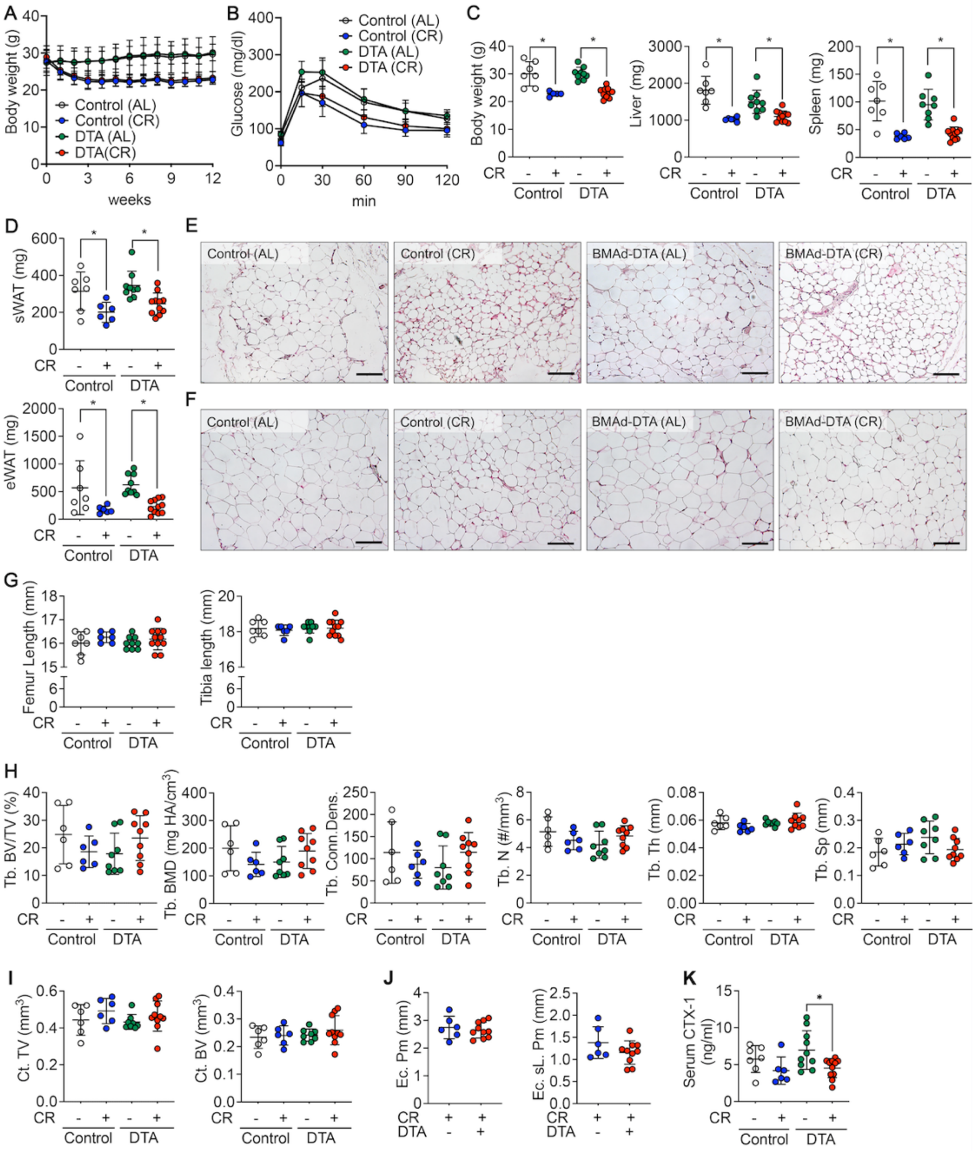

Supplemental Figure 5. Loss of BMAT does not cause alterations to metabolism, or affect trabecular bone in proximal tibiae.

Control and BMAd-DTA male mice at 24 weeks of age underwent 30% CR for 12 weeks. – indicates *ad libitum*, + indicates CR.

A-B. Body weight reduction and glucose tolerance test were recorded before euthanasia.

C-D. Body and soft tissue weights were collected at the end of experiment.

E-F. Representative pictures of subcutaneous (E) and epididymal (F) WAT were collected from paraffin-embedded and H&E-stained sections. Scale bar indicates 100 μm.

G. Femoral and tibial lengths were measured during tissue collection.

H. Trabecular bone parameters in proximal tibiae were determined by μCT scanning. Tb. Trabecular bone; BV/TV: bone volume fraction; BMD: bone mineral density; Conn. Dens: connective density; N: number; Th: thickness; Sp: separation.

I. Distal tibial total volume (TV) and cortical bone volume (BV) were measured by μCT.

J. Calcified distal tibiae were cross-sectioned for dynamic histomorphometry. Endocortical perimeter (Ec. Pm) and single-labelled perimeter (Ec. sL. Pm) were measured by Bioquant software.

K. Serum concentrations of bone resorption marker, CTX-1, were determined by ELISA assay.

Data are presented as mean ± SD. * indicates $P < 0.05$ with two-way ANOVA analyses followed by Šídák's multiple comparisons test.

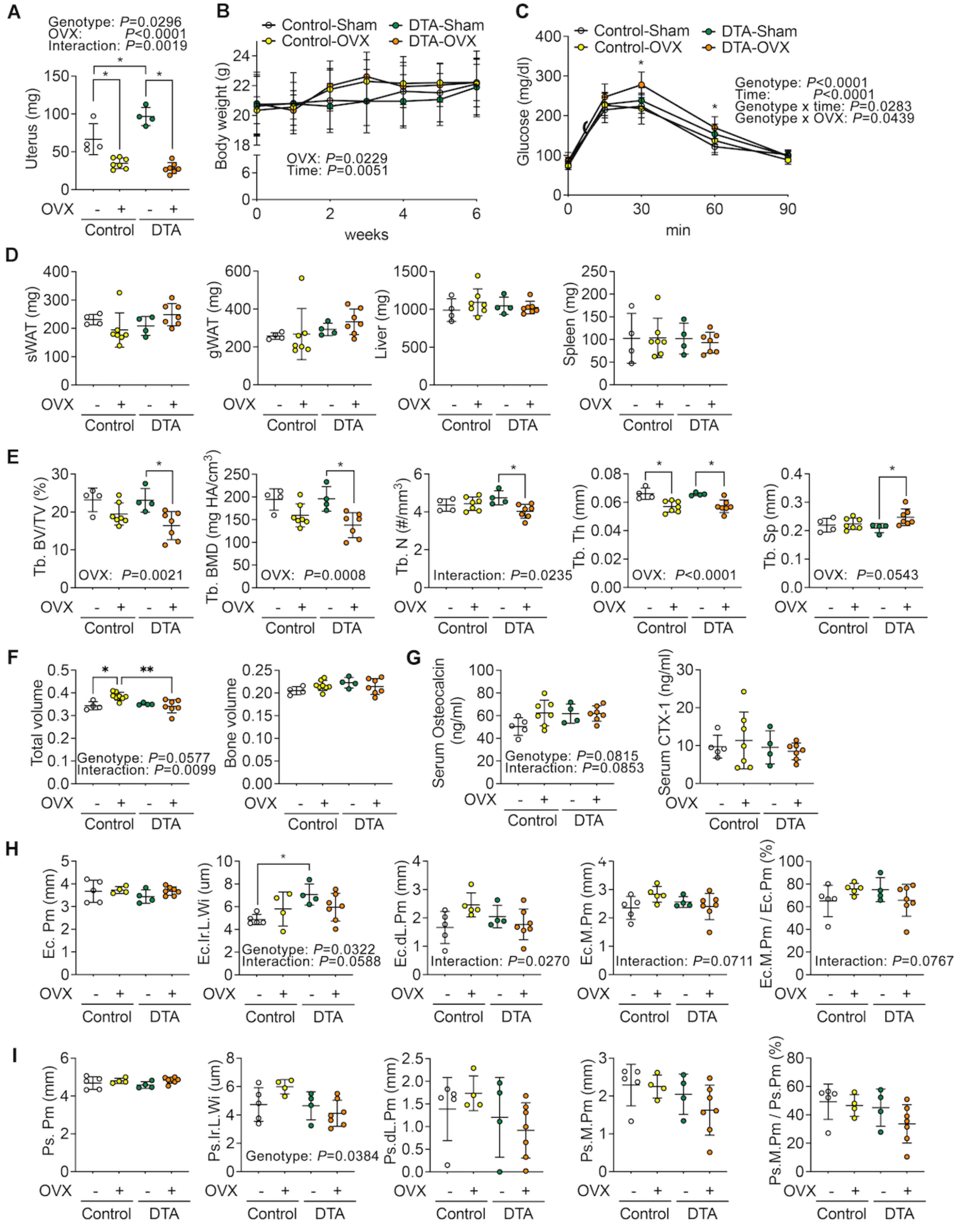
A
Genotype: P=0.0296
OVX: P<0.0001
Interaction: P=0.0019
Uterus (mg)
B
Control-Sham
Control-OVX
DTA-Sham
DTA-OVX
Body weight (g)
OVX: P=0.0229
Time: P=0.0051
weeks
C
Glucose (mg/dl)
Genotype: P<0.0001
Time: P<0.0001
Genotype x time: P=0.0283
Genotype x OVX: P=0.0439
min
D
sWAT (mg)
gWAT (mg)
Liver (mg)
Spleen (mg)
E
Tb. BV/TV (%)
OVX: P=0.0021
Tb. BMD (mg HA/cm3)
OVX: P=0.0008
Tb. N (#/mm3)
Interaction: P=0.0235
Tb. Th (mm)
OVX: P<0.0001
Tb. Sp (mm)
OVX: P=0.0543
F
Total volume
Genotype: P=0.0577
Interaction: P=0.0099
Bone volume
G
Serum Osteocalcin (ng/ml)
Genotype: P=0.0815
Interaction: P=0.0853
Serum CTX-1 (ng/ml)
H
Ec. Pm (mm)
Ec.Ir.L.Wi (um)
Genotype: P=0.0322
Interaction: P=0.0588
Ec.dL.Pm (mm)
Interaction: P=0.0270
Ec.M.Pm (mm)
Interaction: P=0.0711
Ec.M.Pm / Ec.Pm (%)
Interaction: P=0.0767
I
Ps. Pm (mm)
Ps.Ir.L.Wi (um)
Genotype: P=0.0384
Ps.dL.Pm (mm)
Ps.M.Pm (mm)
Ps.M.Pm / Ps.Pm (%)
OVX
Control
DTA

Supplemental Figure 6. BMAd-depletion does not affect body and soft tissue weights following OVX, but impairs glucose metabolism and reduces trabecular bone in proximal tibiae.
Control and BMAd-DTA female mice at 20 weeks of age underwent OVX. Mice were euthanized 6 weeks after surgery. – indicates sham, + indicates OVX.
A-C. Uterine and body weights were measured at the end of study. A glucose tolerance test was performed five weeks after surgery (n=4-7 per group).
D. Soft tissue weights were collected 6 weeks after surgery.
E. Trabecular bone parameters of the proximal tibiae were determined by μCT scanning and analyses. Tb. Trabecular bone; BV/TV: bone volume fraction; BMD: bone mineral density; Conn. Dens: connective density; N: number; Th: thickness; Sp: separation.
F. Cortical bone total volume and bone volume were measured by μCT.
G. Circulating concentrations of osteocalcin and CTX-1 were measured by ELISA.
H-I. Calcein-labelled tibiae were cross-sectioned and used for dynamic histomorphometry. Endocortical (Ec.) and periosteal (Ps.) bone formation parameters were determined by BioQuant software. Pm: perimeter; Ir.L.Wi: inter-label widths; dL. Pm: double-labelled perimeter; M. Pm: mineralizing perimeter; M. Pm / Pm: mineralizing surface proportion.
Data are expressed as mean ± SD. * indicates $P < 0.05$ with three-way (B&C) or two-way ANOVA analyses followed by Šídák's multiple comparisons test.

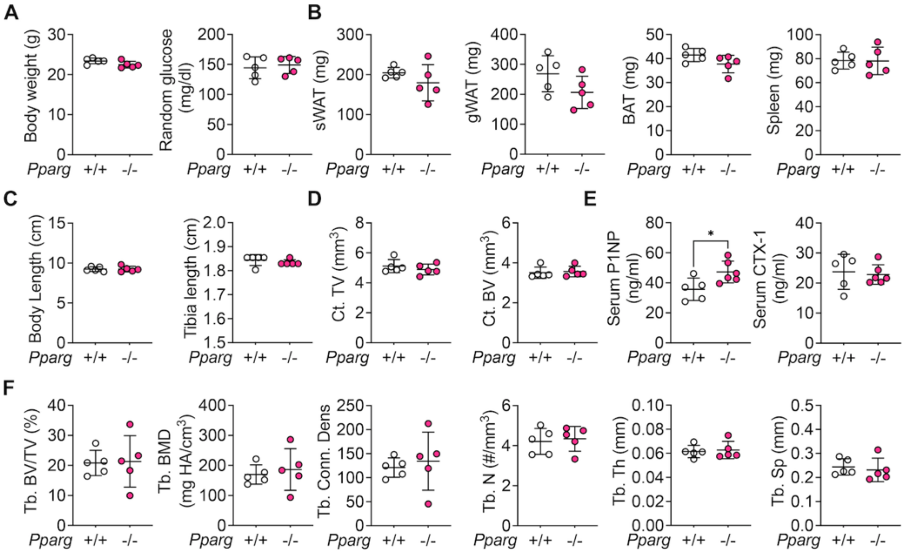

Supplemental Figure 7. BMAd-*Pparg* deficiency does not cause global effects in female mice, and only shows bone phenotypes in BMAd-enriched sites.

Control and BMAd-*Pparg*$^{-/-}$ female mice at 20 weeks of age were euthanized. +/+ indicates control, -/- indicates BMAd-*Pparg* knockout.

A-B. Body weights, random glucose concentrations and soft tissue weights were recorded at the end of study.

C. Length of body from nose to base of tail, and tibiae were measured during tissue collection.

D. Distal tibial cortical bone (Ct.) total volume (TV) and bone volume (BV) were determined by μCT.

E. Circulating concentrations of P1NP and CTX-1 were measured by ELISA.

F. Trabecular bone variables in proximal tibiae were determined by μCT analyses. Tb. Trabecular bone; BV/TV: bone volume fraction; BMD: bone mineral density; Conn. Dens: connective density; N: number; Th: thickness; Sp: separation.

Data are presented as mean ± SD. * indicates $P < 0.05$ with a two-sample t-test.

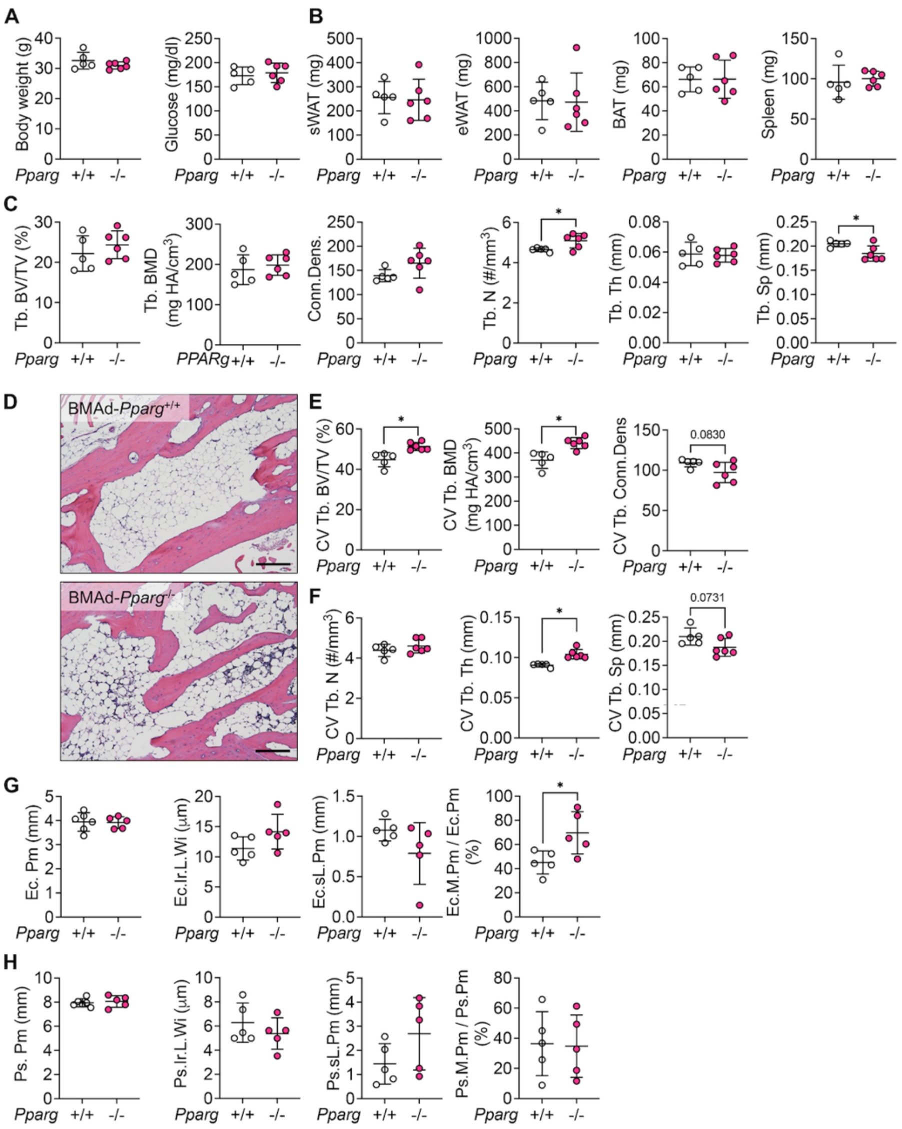

Supplemental Figure 8. BMAd-*Pparg* deficiency does not cause global effects in male mice, and only shows bone phenotypes in BMAd-enriched sites.

Control and BMAd-*Pparg*$^{-/-}$ male mice at 24 weeks of age were euthanized. +/+ indicates control, -/- indicates BMAd-*Pparg* knockout.

A-B. Body weights, random glucose concentrations and soft tissue weights were recorded at the end of study.

C. Trabecular bone variables in proximal tibiae were determined by μCT analyses.

D. Decalcified caudal vertebrae were paraffin-embedded, sectioned and H&E-stained. Representative pictures were taken under 100X magnification. Scale bar indicates 200 μm.

E-F: Trabecular bone parameters in caudal vertebrae were measured by μCT.

Tb. Trabecular bone; BV/TV: bone volume fraction; BMD: bone mineral density; Conn. Dens: connective density; N: number; Th: thickness; Sp: separation.

G-H. Distal tibiae were collected for dynamic histomorphometry. Endocortical (Ec.) and periosteal (Ps.) bone formation parameters were determined by BioQuant software. Pm: perimeter; Ir. L. Wi: inter-label widths; dL. Pm: double-labelled perimeter; M. Pm / Pm: and mineralizing surface proportion.

Data are presented as mean ± SD. * indicates $P < 0.05$ with a two-sample *t*-test. Multiple unpaired *t*-tests were performed in panels C and E-F, and *P* values were adjusted for multiple comparisons using two-stage step-up (Benjamini, Krieger, and Yekutieli) with FDR method.
